# Breaching the Bridge: An Investigation into Doctor-Patient Miscommunication as a Significant Factor in the Violence against Healthcare Workers in Palestine

**DOI:** 10.1155/2021/9994872

**Published:** 2021-07-23

**Authors:** Munther Saeedi, Nihad Al-Othman, Maha Rabayaa

**Affiliations:** ^1^Language Centre/Faculty of Human Science, An-Najah National University, Nablus, State of Palestine; ^2^Faculty of Medicine and Health Sciences, An-Najah National University, Nablus, State of Palestine

## Abstract

**Background:**

Workplace violence is a common issue worldwide that strikes all professions, and healthcare is one of the most susceptible ones. Verbal and nonverbal miscommunications between healthcare workers and patients are major inducers for violent attacks.

**Aim:**

To study the potential impact of verbal and nonverbal miscommunications between the patients and healthcare workers upon workplace violence from the patients' perspectives.

**Methods:**

A descriptive cross-sectional study was performed from November to December 2020. Patients and previously hospitalized patients were asked to complete a self-reported questionnaire that involved items of verbal and nonverbal miscommunication. With the use of a suitable available sample composed of 550 participants, 505 had completed the questionnaire and were included in the study. The data were analyzed by using SPSS version 22 software.

**Results:**

7.2% of the study population reported participating in nonverbal violence and 19.6% participated in verbal violence against healthcare workers. The nonverbal and verbal violence was characteristically displayed by the patients who are male, younger than 30 years old, and bachelor's degree holders. The results of the study demonstrated that the verbal and nonverbal miscommunications between the patients and healthcare workers were the major factors in provoking violent responses from patients. Factors, such as age, gender, and level of education, were significant indicators of the type of patients who were more likely to respond with violence.

**Conclusion:**

Workplace violence, either verbal or nonverbal, in the health sector is a public health concern in Palestine. The verbal and nonverbal communication skills of healthcare workers should be developed well enough to overcome the effect of miscommunication provoking violent acts from patients and their relatives as well.

## 1. Introduction

The National Institute for Occupational Safety and Health (NIOSH) defines workplace violence as “ any physical assault, threatening behavior, or verbal abuse occurring in the work setting” [1]. Globally, workplace violence has gained a greater concern in the recent century. Assaults and acts of violence were observed against all professionals irrespective of the nature of their profession, and the healthcare professional is not an exception. However, it has been reported that retailing and service sector encounter more than 80% of workplace violence in the United State. And the health sector workers encounter workplace violence sixteen times more than workers in any other service sector [2]. Violent attacks against healthcare workers abound in clinics, health care centers, and hospitals; every day, the media shows something related to violence against healthcare workers around the world. Several factors, including individual, organizational, and environmental factors, are the likely origins of the various forms of violence in the healthcare sector [3]. Unfortunately, the precise incidence of workplace violence globally is not documented, especially in developing countries. However, workplace violence is negatively affecting work performance since it is associated with decreased productivity, decreased morale, increased stress and depression, and lower service efficiency among employees [4]. Healthcare workers, irrespective of where they work, are very likely to be abused verbally and physically, which may result in disappointment, despair, and in certain circumstances, frustration among them [5]. Healthcare workers, in general, and doctors, in specific, are always targeted by patients or patients' relatives; doctors serving in Accident and Emergency Departments are more likely to be victims of violent attacks by patients and relatives more than any other healthcare workers [6].

Patient-healthcare worker communication is a central clinical requirement, and it is taken for granted that the success of healthcare workers is no longer attributed to their capacity to provide health care and medical services; neither is it related to how much information they have. It depends, to a large extent, on their ability to communicate with their clients and their family members [7]. A healthcare worker is expected to be a good communicator; otherwise, s/he is likely to be assaulted and attacked by patients or their relatives due to dissatisfaction with the health service provided [8, 9]. Recently, health care workers have been victims of clients' assaults and violence, whether it is verbal or nonverbal [10, 11]. Acts of violence against healthcare workers can be attributed to several factors including, but not limited to, long waiting periods, dissatisfaction with prescriptions and treatment methods, disagreement with doctors, verbal offenses or negative comments, and the negative impact of certain medications, such as recreational drugs [12]. A large bulk of these incidents may be attributed to a lack of good communication skills that is required of healthcare workers in order to put their patients at ease before commencing their medical and physical examination [7, 13].

Most of the previous studies have focused on the incidence of workplace violence from the workers' perspective. This study is a leading one in Palestine as it shows the incidence of workplace violence from the patients' perspectives. This study also aims to identify the crucial communication skills, verbal or nonverbal, that should be incorporated in the communications curriculum to explore how communication lapses may lead to the occurrence of violent attacks against doctors.

## 2. Materials and Methods

### 2.1. Ethical Consideration

This study received official ethical approval from the Institutional Review Board at An-Najah National University located in Nablus/Palestine. The study abided by “the Declaration of Helsinki (DOH).” All ethical considerations for medical research concerning human subjects were enforced. The human subject confidentiality and rights were preserved throughout the study. Written informed consent was provided and handed to each patient (Appendix). The form described the study procedure, duration, benefit, and lack of any harmful intentions. Moreover, the form indicated that all data collected would be used for research purposes only, while any information related to the patient would be kept confidential from all parties except the research investigators. The patients were fully informed that participation in the study was voluntary and that no penalty would be enforced in case of nonparticipation.

### 2.2. Study Sample

A cross-sectional study was carried out from November to December of the academic year 2020/2021 on patients attending hospitals seeking medical service, e.g., clinics and laboratories, surgery operations, and emergency rooms to investigate the doctor-patient miscommunication as a significant factor in violence against healthcare workers in Palestine before discharge and during follow-up visits. A convenient nonprobability available sample took part in this study. The sample size was estimated using the Jekel equation. The assumption of the probability of violence against healthcare workers was 0.5 with a confidence level of 95%; the estimated minimum sample size was 384. Nevertheless, the researchers decided to increase the sample size to 550, to decrease the standard error of the mean and to account for the nonresponse rate. In the end, 505 participants, who were previously hospitalized in seven hospitals with different specialties in Palestine, completed the questionnaire and were included in the study.

### 2.3. Inclusion and Exclusion Criteria

The inclusion criteria included patients or previously hospitalized patients within six months of questionnaire administration and agreed to participate in this study. The patients were from different age groups, residential areas (city, camp, or village), and levels of education. The exclusion criteria included patients who refused to participate in the study and the doctors who work in the medical field.

### 2.4. Study Instrument

A self-administered questionnaire in Arabic was used for data collection and was distributed to the study population. The questionnaire was made up of four sections: sociodemographic factors including age, level of education, gender, and place of residence, verbal miscommunication section which comprised 14 items, the nonverbal miscommunication section which was composed of 6 items, and two questions whether a patient had ever participated in verbal or nonverbal violence. To ensure the validity of the study instrument, the tool was given to five experts in the field of public health. There was an agreement among them regarding the content of the questionnaire.

### 2.5. Pilot Study

A pilot study was performed on 30 individuals from different age groups to determine questionnaire wording, formatting, completeness of responses, clarity of choices, the relevance of the statements, and the time needed to fill the form. The questionnaire was modified accordingly. The internal consistency of the questionnaire was measured based on Cronbach Alpha values (0.81) before data collection.

### 2.6. Statistical Analysis

All statistical analyses were conducted using Statistical Package for the Social Sciences version 22 (SPSS 22). Descriptive analyses were used for sociodemographic characteristics. An initial univariate analysis was used to compare sociodemographic variables and variables related to exposure to violence. Chi-Square Test was used to determine the relationship between sociodemographic variables and verbal and nonverbal miscommunications. A *p* value of <0.05 was considered statistically significant.

## 3. Results

### 3.1. Demographic Characteristics of the Study Population

The data were analyzed and tested for normality and found to be normally distributed. Of the 505 patients who took part in the study, 272 (53.9%) were males, and 233 (46.1%) were females. The age group ≤29 years was the highest 241 (47.7%), while the age group 50-59 interval 45 (8.9%) was the lowest. According to the level of education, the bachelor's degree was the highest 299 (59.2%), while the diploma was the lowest 34 (6.7%). Based on the place of residence, 205 (49.9%) of the study population were from villages, and 48 (9.5%) of them were from camps Table 1.

### 3.2. The Distribution of Physical and Verbal Violence against Healthcare Workers

The researchers found that the total percentage of patients involved in physical and verbal violence against healthcare workers was 26.8%; 7.2% were involved in the act of physical violence; 4% of them were males while 3.2% were females, 4.2% were ≤29 years old, and 4.6% were bachelor's degree holders. On the other hand, 19.6% of the study population were involved in verbal violence against healthcare workers; 13% were males while 6.6% were females, 9.6% were less than 30 years old, and 13% were bachelor's degree holders (Table 2). Of the study population, 73.2% were not involved in any act of violence against healthcare workers.

### 3.3. Verbal Miscommunications in relation to Different Demographic Factors

The ratios and correlations between the 14 verbal miscommunication items and the different demographic factors from the patient's perspective are found in Table 3. It was revealed that most of the study population agreed that violence, physical or verbal, against healthcare workers was due to inappropriate verbal communication between healthcare providers and patients, based on the evaluated parameters (see Table 3). The reasons for violence, either physical or verbal, against healthcare workers are mostly because the healthcare workers: do not use simplified, clear language when they communicate with patients and their relatives (63.5%), do not consider patients and their relatives' level of education (77.1%), do not speak clearly when they communicate with patients and their relatives (74.8%), do not take into consideration the psychological state of patients and their relatives (79.8%), do not pick the right time to break bad news (54.9%), do not answer patients' and relatives' questions well (75.3%), show some superiority when communicating with patients and relatives (73.7%), do not show sympathy and empathy when communicating with patients and relatives (72.7%), do not focus when communicating with patients and relatives (76.6%), do not use courteous language when communicating with patients and relatives (64.7%), are not competent enough to ask the right questions when communicating with patients and relatives (42%), do not listen attentively when communicating with patients and relatives (72.3%), do not handle patients' and relatives' complaints appropriately (71.7%), and do not ask open-ended questions competently to enable patients and their relatives to speak freely (68.9%).

The role of various verbal miscommunications in initiating workplace violence is found to be significantly variable based on the patient's characteristics. Significant differences were found between male and female responses regarding these items: describing the language used by healthcare workers when dealing with patients and their families (*p* < 0.05), the proper time for healthcare workers to break bad news (*p* < 0.01), whether healthcare workers answer all the questions raised by patients and their families (*p* < 0.05), and whether healthcare workers communicate courteously with patients and their families (*p* < 0.001) (Table 3).

Significant differences were found between the responses of the different age groups regarding these items: healthcare workers do not use simplified clear language (*p* < 0.01), do not speak clearly when they communicate with patients and their relatives (*p* < 0.05), and do not use courteous language when communicating with patients and relatives (*p* < 0.01) (Table 3). According to the level of education, a significant difference was found regarding the item that healthcare workers cannot handle patients' and relatives' complaints appropriately (*p* < 0.05). There is no significant difference between the place of residence and their answers (Table 3).

### 3.4. Nonverbal Miscommunications in relation to Different Demographic Factors

The ratios and correlations between the six nonverbal miscommunication items and the different demographic factors from the patient's perspective are found in Table 4. Patients and previously hospitalized patients are influenced greatly by several nonverbal miscommunications. The reasons for violence, either physical or verbal, against healthcare workers are mostly because the healthcare workers: do not maintain good eye contact (66.6%), do not smile frequently (64.5%), do not have a comfortable voice tone (70.7%), often have a frown on their faces (47.9%), are often seated provocatively (71.9%), and do not employ handshakes properly (49.9%) (Table 4).

Significant differences were found between male and female responses to three items: healthcare workers do not maintain good eye contact (*p* < 0.05), they have a frown when communicating with patients and their families (*p* < 0.01), and they do not employ handshakes properly (*p* < 0.05). Furthermore, based on the level of education, a significant difference was found in answers regarding the item stating that the healthcare workers are often seated provocatively (*p* < 0.001) (Table 4).

## 4. Discussion

To the knowledge of the researchers, this is the first study in Palestine describing the violence against healthcare workers from the patients' perspectives. This study revealed that 7.2% of the study population was involved in an act of physical violence against healthcare workers. Also, 19.6% of the study population was involved in an act of verbal violence against healthcare workers. In Palestine, violence against healthcare workers was 20.8% nonverbal and 59.6% verbal violence from the view of the healthcare workers [11, 14]. In another study, 35.6% of the healthcare workers in the emergency department were exposed to nonverbal violence, while 71.2% of them were exposed to verbal violence [14]. In Jordan, 10.4% of violence against healthcare workers was nonverbal, while 63.5% of violence was verbal [15]. A similar study in Saudi Arabia revealed that 5.3% of the violence against healthcare workers was nonverbal while 39.2% was verbal [16]. The verbal form of violence was the most dominant form of violence against healthcare workers with generally high rates of violence reported from the healthcare workers' perspectives [15–18]. However, rare studies are available about workplace violence in the health sector from the patient perspective. A study conducted in China reported that 1.5% of patients responded to medical disputes by resorting to violence against healthcare workers. Significantly, in the reports of violence against healthcare workers, it was found that such assaults were more likely to be carried out by male patients, patients with a high-income level, and patients generally dissatisfied with life. On the other hand, it was established that trust between the healthcare worker and patient resulted in nonviolent resolutions of medical disputes [19].

It can be observed that the percentage of violence is greatly variable when it is studied from the perspective of either patients or healthcare workers. These controversial results from different perspectives in the rate of workplace violence in the health sector affirm the need for definitive policies regarding the definition of violence, proper reporting strategies, and actions to control this prevalent problem with its detrimental impact on the effectiveness of healthcare service, medical practitioner psychology, and patient satisfaction [20, 21].

The optimal health service requires effective communication between the patient and the healthcare workers, whether in the verbal or nonverbal form [22]. This study focuses on different parameters related to both forms of communication between the patients and healthcare workers from the patients' perspective since patient satisfaction has a critical role in the development of the healthcare sector and the reduction of potential acts of violence against the healthcare workers [23]. The personal interaction between healthcare workers and patients is a pivotal requirement to achieve an effective medical service and to avoid adverse outcomes. Consequently, the disruption of this complex communication, either verbal or nonverbal, is a vital reason for the violence in the health sector, in addition to other organizational, environmental, and individual factors, such as long waiting time, the discrepancy between patients' expectations and services received, psychiatric conditions, and insufficient security [24, 25].

In this study, fourteen items involved in the verbal miscommunication between the patient and the healthcare workers have been evaluated from the patient's perspective (Table 3). This study revealed that most of the study population agreed that violence, physical or verbal, against healthcare workers was due to inappropriate verbal communication between healthcare providers and patients. The results of this study are consistent with another recent study in which ineffective communications, poor experience, and other socio-behavioral problems were shown to be the major factors contributing to workplace violence [26]. A previous study reported that effective management of workplace violence against healthcare providers requires training courses that aid in constructing healthcare worker-patient relationships, improving the healthcare workers' verbal and nonverbal communication skills, and accurate reporting of each violent incident [27–30].

The variables of gender, age, and level of education have been found to influence a patient's propensity to a violent response to miscommunication with a healthcare worker (Table 3). These variations have been previously identified as risk factors contributing to workplace violence; healthcare workers are at greater risk of assault from young male patients with a low level of education, in addition to other societal, organizational, and patient- and doctor-related factors [31–34].

Workplace violence against healthcare workers has deleterious effects on the psycho-social well-being of the providers, as well as on patient management [35, 36]. As a result, healthcare workers need to take into consideration the patient's variables such as age, gender, and level of education during verbal communication to decrease any potential for violent attacks against them. This also implies the importance of training courses for healthcare workers in proper communications, including verbal or nonverbal skills, with patients as a prepractical requirement [37].

By evaluating the role of six items of nonverbal communication as a reason for violence against healthcare workers (Table 4), the patients' gender and level of education were found to have significant influence. The results of this study are consistent with what was previously reported as important but overlooked nonverbal communication lapses in patient-doctor communication [37, 38]. Nonverbal communication can foster trust between patient and doctor [39]. Effective verbal and nonverbal communication in the workplace is the first line of defense against violence, as good communication skills will make the healthcare workers more confident in thwarting aggressive attacks [40, 41].

## 5. Conclusion and Recommendations

In conclusion, workplace violence against healthcare workers is an increasing problem in the health sector. As effective communication is vital in achieving good healthcare, patient satisfaction, staff confidence, and staff rights, the verbal and nonverbal miscommunications between the patients and healthcare workers are a serious concern because of their adverse impact upon the integrity of the medical services. Health care workers should take into consideration the variations in patients' age, gender, level of education, and place of residence in order to communicate effectively and to avoid the possibility of violent confrontations. The improvement of both verbal and nonverbal communication skills among healthcare workers is recommended to foster the proper level of trust between patients and their healthcare providers. This requires extensive training courses as a prepractical requirement. Finally, it is important to develop standard policies about the definition of workplace violence, reporting methods and to put proper penalties in place that protect the rights of all involved parties in the conflict.

## 6. Limitations of the Study

The patients who refused to participate in the study could be the ones who might be a greater contributor to the violence against healthcare workers. As this research is the first of its kind in Palestine, there are no previous studies in the area available for the comparison of data. The geographic and demographic variations between patients in more such studies would provide wider-ranging findings. Moreover, there are no definitive strategies regarding workplace violence in the health sector to use as a baseline in violence classification and required actions.

## Figures and Tables

**Table 1:** Demographic characteristics of the study population (*n* = 505).

Variable	Number	Percentage (%)
Gender		
Male	272	53.9
Female	233	46.1
Total	505	100
Age groups (years)		
≤29	241	47.7
30-39	125	24.8
40-49	94	18.6
50-59	45	8.9
Total	505	100
Level of education		
Tawjihi or less	35	6.9
Diploma	34	6.7
Bachelor^∗^	299	59.2
Graduated studies^∗∗^	137	27.1
Total	505	100
Place of residence		
City	205	40.6
Camp	48	9.5
Village	252	49.9
Total	505	100

^∗^undergraduate; ^∗∗^completed graduation.

**Table 2:** Distribution of patients or previously hospitalized patients involved in physical or verbal violence against healthcare workers (*n* = 505).

Item	The total %	Gender		Age groups				Level of education			
		Male %	Female %	≤29%	30-39%	40-49%	50-59%	Tawj%	Diplo%	Ba%	GS%
Individuals involved in an act of physical violence against healthcare workers	7.2	4	3.2	4.2	2	1	0	0.8	0.4	4.6	1.4
Individuals involved in an act of verbal violence against healthcare workers	19.6	13	6.6	9.6	5.4	4	0.6	1.6	1.4	13	3.6

Tawj: Tawjihi (higher secondary school); Diplo: Diploma; Ba: Bachelor; GS: Graduate studies.

**Table 3:** Verbal miscommunications in relation to different demographic factors (*n* = 505).

Item	Strongly agree (%)	Agree (%)	I do not know (%)	Disagree (%)	Strongly disagree (%)	Gender (*p* value)	Age groups (*p* value)	Level of education (*p* value)	Place of residence (*p* value)
(1) One of the reasons for violence, physical or verbal, against healthcare workers is because they do not use simplified, clear language	16.2	47.3	11.1	18.8	6.5	0.017^∗^	0.004^∗∗^	0.056	0.899
(2) One of the reasons for violence, physical or verbal, against healthcare workers is because they do not consider patients and their relatives' educational level	22.6	54.5	6.1	12.3	4.6	0.953	0.337	0.05^∗^	0.921
(3) One of the reasons for violence, physical or verbal, against healthcare workers is because they do not speak clearly when they communicate with patients and their relatives	21.4	52.7	8.1	14.1	3.8	0.247	0.033^∗^	0.584	0.779
(4) One of the reasons for violence, physical or verbal, against healthcare workers is because they do not take into consideration the psychological status of patients and their relatives	29.9	49.9	5.9	10.7	3.6	0.753	0.914	0.097	0.135
(5) One of the reasons for violence, physical or verbal, against healthcare workers is because they do not pick the right time to break the bad news	13.1	41.8	18.2	21.8	5.1	0.009^∗∗^	0.643	0.648	0.706
(6) One of the reasons for violence, physical or verbal, against healthcare workers is because they do not answer patients and relatives' questions well	25	50.3	8.7	11.7	4.4	0.042^∗^	0.213	0.511	0.948
(7) One of the reasons for violence, physical or verbal, against healthcare workers is because they show some superiority when communicating with patients and relatives	32.7	41	7.1	12.3	6.9	0.308	0.714	0.850	0.603
(8) One of the reasons for violence, physical or verbal, against healthcare workers is because they do not show sympathy and empathy when communicating with patients and relatives	23.4	49.3	8.1	15.6	3.6	0.998	0.093	0.598	0.236
(9) One of the reasons for violence, physical or verbal, against healthcare workers is because they do not show much concentration when communicating with patients and relatives	27.7	48.9	9.1	10.3	4	0.162	0.238	0.934	0.837
(10) One of the reasons for violence, physical or verbal, against healthcare workers is because they do not use courteous language when communicating with patients and relatives	17.8	46.9	11.5	18.4	5.3	0.001^∗∗^	0.003^∗∗^	0.417	0.075
(11) One of the reasons for violence, physical or verbal, against healthcare workers is because they are not competent enough to ask the right questions when communicating with patients and relatives	8.1	33.9	23.4	28.9	5.7	0.159	0.316	0.288	0.432
(12) One of the reasons for violence, physical or verbal, against healthcare workers is because they do not listen attentively when communicating with patients and relatives	17.4	54.9	9.3	14.1	4.4	0.536	0.428	0.797	0.974
(13) One of the reasons for violence, physical or verbal, against healthcare workers is because they cannot handle patients and relatives' complaints appropriately	17	54.7	11.9	11.9	4.6	0.840	0.367	0.027^∗^	0.887
(14) One of the reasons for violence, physical or verbal, against healthcare workers is because they are not competent enough to ask open-ended questions to enable patients and their relatives to speak freely	17.4	51.5	13.1	14.7	3.4	0.129	0.305	0.863	0.926

^∗^ *p* < 0.05, ^∗∗^*p* < 0.01, ^∗∗∗^*p* < 0.001.

**Table 4:** Nonverbal miscommunications in relation to different demographic factors (*n* = 505).

Item	Strongly agree	Agree	I do not know	Disagree	Strongly disagree	Gender (*p* value)	Age groups (*p* value)	Level of education (*p* value)	Place of residence (*p* value)
(1) One of the reasons for violence, physical or verbal, against healthcare workers is because they do not maintain good eye contact	13.3	53.3	16.4	13.7	3.4	0.05^∗^	0.079	0.065	0.140
(2) One of the reasons for violence, physical or verbal, against healthcare workers is because they do not smile frequently	17.8	46.7	8.9	20.6	5.9	0.228	0.617	0.081	0.418
(3) One of the reasons for violence, physical or verbal, against healthcare workers is because they do not have a comfortable voice tone	18.8	51.9	10.3	14.7	4.4	0.195	0.210	0.076	0.402
(4) One of the reasons for violence, physical or verbal, against healthcare workers is because they often have a frown on their faces	11.9	36	18.4	27.5	6.1	0.003^∗∗^	0.433	0.086	0.827
(5) One of the reasons for violence, physical or verbal, against healthcare workers is because they are often seated in a provocatively	20.8	51.1	9.9	14.1	4.2	0.107	0.377	0.001^∗∗∗^	0.809
(6) One of the reasons for violence, physical or verbal, against healthcare workers is because they do not employ handshakes properly	16.2	33.7	15	26.9	8.1	0.05^∗^	0.481	0.494	0.497

^∗^ *p* < 0.05, ^∗∗^*p* < 0.01, ^∗∗∗^*p* < 0.001.

## Data Availability

All the utilized data to support the findings of the current study are included in the article.

## Supplementary Materials

Supplementary Materials All the utilized data to support the findings of the current study are included in the supplementary material.
