# Supplementary material for: Breaching the Bridge: An Investigation into Doctor-Patient Miscommunication as a Significant Factor in the Violence against Healthcare Workers in Palestine
Source: Biomed Res Int. 2021 Jul 23;2021:9994872. doi: 10.1155/2021/9994872 (PMC8324345; doi:10.1155/2021/9994872)
Supplement: Supplementary Materials — All the utilized data to support the findings of the current study are included in the supplementary material. [file 9994872.f1.zip › Raw data Dr Nihad.pdf]

Sex	Age groups	Level of education	Place of residence
Male	less than 50 years	Graduate studies	Village
Male	less than 50 years	Graduate studies	Village
Male	less than 30 years	Graduate studies	City
Female	less than 30 years	Graduate studies	City
Female	less than 50 years	Graduate studies	City
Female	less than 30 years	Graduate studies	City
Male	less than 40 years	Bachelor degree	Village
Female	less than 30 years	Bachelor degree	Village
Female	less than 30 years	Bachelor degree	City
Female	less than 30 years	Bachelor degree	Village
Female	less than 40 years	Bachelor degree	Village
Female	less than 30 years	Bachelor degree	City
Female	less than 40 years	Graduate studies	City
Male	less than 50 years	Bachelor degree	Camp
Female	less than 30 years	Graduate studies	City
Female	less than 30 years	Bachelor degree	City
Female	less than 30 years	Graduate studies	City
Female	less than 30 years	Tawjihi or less	Village
Male	less than 30 years	Graduate studies	City
Female	less than 30 years	Bachelor degree	City
Female	less than 30 years	Bachelor degree	Village
Female	less than 30 years	Bachelor degree	Village
Female	less than 30 years	Bachelor degree	Village
Male	less than 30 years	Bachelor degree	City
Male	less than 30 years	Bachelor degree	City
Female	less than 30 years	Bachelor degree	City
Female	less than 30 years	Bachelor degree	City
Female	less than 30 years	Bachelor degree	Village
Female	less than 30 years	Bachelor degree	City
Female	less than 30 years	Bachelor degree	City
Female	less than 30 years	Bachelor degree	Village
Female	less than 30 years	Bachelor degree	City
Male	less than 30 years	Bachelor degree	Village
Female	less than 30 years	Bachelor degree	Village
Male	less than 40 years	Bachelor degree	Village
Male	less than 40 years	Bachelor degree	Village
Male	less than 50 years	Bachelor degree	Village
Male	less than 40 years	Bachelor degree	City
Male	less than 60 years	Bachelor degree	City
Male	less than 40 years	Graduate studies	City
Male	less than 40 years	Graduate studies	Village
Female	less than 40 years	Graduate studies	Village
Male	less than 30 years	Tawjihi or less	Village
Male	less than 30 years	Bachelor degree	Village
Male	less than 30 years	Bachelor degree	Village
Female	less than 40 years	Bachelor degree	Village
Female	less than 40 years	Bachelor degree	Village
Female	less than 50 years	Graduate studies	City

Male	less than 40 years	Graduate studies	City
Female	less than 40 years	Bachelor degree	Village
Female	less than 30 years	Bachelor degree	Village
Male	less than 40 years	Bachelor degree	Village
Male	less than 50 years	Diploma	Camp
Male	less than 60 years	Bachelor degree	City
Male	less than 40 years	Bachelor degree	City
Male	less than 60 years	Graduate studies	Camp
Female	less than 50 years	Graduate studies	Village
Female	less than 40 years	Graduate studies	City
Male	less than 60 years	Bachelor degree	Camp
Female	less than 40 years	Diploma	Village
Female	less than 50 years	Graduate studies	Village
Female	less than 40 years	Graduate studies	City
Male	less than 40 years	Bachelor degree	Village
Male	less than 60 years	Bachelor degree	Village
Male	less than 40 years	Tawjihi or less	Village
Male	less than 50 years	Bachelor degree	Village
Male	less than 50 years	Diploma	Camp
Female	less than 50 years	Graduate studies	Camp
Male	less than 30 years	Bachelor degree	Village
Female	less than 50 years	Bachelor degree	Village
Female	less than 30 years	Bachelor degree	Village
Male	less than 50 years	Graduate studies	Camp
Female	less than 30 years	Bachelor degree	Village
Male	less than 40 years	Graduate studies	City
Male	less than 30 years	Bachelor degree	City
Female	less than 60 years	Bachelor degree	City
Male	less than 30 years	Bachelor degree	Village
Female	less than 30 years	Bachelor degree	Village
Male	less than 40 years	Graduate studies	Village
Male	less than 30 years	Bachelor degree	Village
Male	less than 60 years	Bachelor degree	City
Female	less than 40 years	Graduate studies	City
Female	less than 30 years	Diploma	Village
Female	less than 40 years	Bachelor degree	City
Male	less than 40 years	Diploma	Village
Female	less than 30 years	Graduate studies	City
Male	less than 60 years	Graduate studies	City
Male	less than 30 years	Bachelor degree	City
Male	less than 30 years	Bachelor degree	Village
Male	less than 30 years	Graduate studies	Village
Male	less than 50 years	Graduate studies	Village
Female	less than 30 years	Bachelor degree	Village
Male	less than 30 years	Bachelor degree	Village
Male	less than 50 years	Graduate studies	Village
Male	less than 50 years	Tawjihi or less	Village
Male	less than 30 years	Bachelor degree	Village
Female	less than 30 years	Bachelor degree	Village
Male	less than 30 years	Bachelor degree	Village
Male	less than 40 years	Bachelor degree	Village
Male	less than 40 years	Bachelor degree	Village
Male	less than 30 years	Bachelor degree	City
Male	less than 40 years	Bachelor degree	City
Male	less than 60 years	Graduate studies	Village

Female	less than 30 years	Bachelor degree	Village
Female	less than 40 years	Graduate studies	City
Male	less than 40 years	Graduate studies	Village
Female	less than 30 years	Bachelor degree	Village
Male	less than 30 years	Bachelor degree	Camp
Female	less than 40 years	Bachelor degree	City
Male	less than 40 years	Bachelor degree	Village
Male	less than 30 years	Tawjihi or less	Village
Male	less than 50 years	Graduate studies	Village
Male	less than 30 years	Bachelor degree	Village
Male	less than 50 years	Bachelor degree	City
Male	less than 50 years	Graduate studies	City
Male	less than 50 years	Bachelor degree	City
Female	less than 30 years	Bachelor degree	Village
Male	less than 40 years	Bachelor degree	Village
Male	less than 50 years	Tawjihi or less	City
Female	less than 30 years	Bachelor degree	Village
Female	less than 30 years	Bachelor degree	Village
Male	less than 50 years	Graduate studies	Camp
Male	less than 30 years	Bachelor degree	Village
Male	less than 30 years	Bachelor degree	Village
Female	less than 30 years	Bachelor degree	Village
Female	less than 30 years	Diploma	Village
Male	less than 40 years	Bachelor degree	Village
Male	less than 50 years	Bachelor degree	Village
Female	less than 30 years	Graduate studies	City
Female	less than 30 years	Bachelor degree	Village
Male	less than 40 years	Bachelor degree	Village
Female	less than 40 years	Bachelor degree	Village
Female	less than 30 years	Bachelor degree	Village
Male	less than 60 years	Graduate studies	City
Male	less than 50 years	Graduate studies	City
Male	less than 40 years	Tawjihi or less	Village
Male	less than 30 years	Bachelor degree	City
Male	less than 40 years	Tawjihi or less	Village
Male	less than 40 years	Diploma	City
Male	less than 30 years	Tawjihi or less	Village
Male	less than 30 years	Tawjihi or less	Village
Female	less than 30 years	Graduate studies	Village
Female	less than 30 years	Bachelor degree	Village
Male	less than 30 years	Bachelor degree	City
Female	less than 40 years	Bachelor degree	City
Female	less than 50 years	Graduate studies	Village
Female	less than 40 years	Graduate studies	City
Male	less than 50 years	Graduate studies	City
Female	less than 30 years	Bachelor degree	Village
Male	less than 40 years	Bachelor degree	Village
Female	less than 30 years	Bachelor degree	Village
Male	less than 40 years	Graduate studies	Village
Female	less than 40 years	Bachelor degree	City
Male	less than 50 years	Bachelor degree	City
Male	less than 30 years	Bachelor degree	Village
Male	less than 60 years	Graduate studies	Village
Male	less than 40 years	Bachelor degree	Village
Female	less than 30 years	Bachelor degree	Camp

Female	less than 40 years	Bachelor degree	City
Male	less than 50 years	Graduate studies	Village
Female	less than 50 years	Graduate studies	City
Male	less than 60 years	Graduate studies	City
Female	less than 40 years	Bachelor degree	City
Female	less than 30 years	Bachelor degree	City
Female	less than 30 years	Bachelor degree	Village
Female	less than 30 years	Bachelor degree	City
Male	less than 40 years	Bachelor degree	Village
Female	less than 30 years	Diploma	Village
Female	less than 40 years	Graduate studies	Village
Female	less than 30 years	Bachelor degree	Camp
Female	less than 30 years	Bachelor degree	City
Male	less than 60 years	Graduate studies	City
Male	less than 50 years	Graduate studies	City
Male	less than 40 years	Diploma	Village
Male	less than 30 years	Tawjihi or less	Village
Male	less than 30 years	Graduate studies	Village
Male	less than 50 years	Graduate studies	Village
Female	less than 30 years	Bachelor degree	City
Male	less than 50 years	Graduate studies	City
Female	less than 40 years	Graduate studies	City
Female	less than 40 years	Bachelor degree	City
Female	less than 40 years	Bachelor degree	City
Female	less than 30 years	Bachelor degree	City
Female	less than 40 years	Bachelor degree	Camp
Female	less than 30 years	Bachelor degree	City
Male	less than 30 years	Diploma	Village
Female	less than 30 years	Bachelor degree	Village
Male	less than 50 years	Graduate studies	Camp
Female	less than 30 years	Graduate studies	Village
Female	less than 30 years	Bachelor degree	Village
Male	less than 40 years	Tawjihi or less	Village
Female	less than 40 years	Bachelor degree	Village
Male	less than 60 years	Bachelor degree	City
Male	less than 40 years	Graduate studies	Village
Female	less than 30 years	Bachelor degree	Village
Female	less than 30 years	Bachelor degree	Village
Male	less than 40 years	Tawjihi or less	Village
Male	less than 50 years	Graduate studies	Village
Male	less than 60 years	Bachelor degree	Village
Male	less than 40 years	Bachelor degree	Village
Male	less than 60 years	Bachelor degree	Village
Male	less than 60 years	Bachelor degree	Village
Male	less than 40 years	Bachelor degree	Village
Male	less than 40 years	Graduate studies	Camp
Female	less than 30 years	Tawjihi or less	Camp
Male	less than 30 years	Bachelor degree	Camp
Male	less than 50 years	Bachelor degree	Camp
Male	less than 30 years	Tawjihi or less	Camp
Male	less than 50 years	Diploma	Village
Male	less than 50 years	Bachelor degree	Camp
Female	less than 30 years	Diploma	Camp
Male	less than 30 years	Bachelor degree	Camp
Female	less than 30 years	Bachelor degree	Camp

Female	less than 30 years	Bachelor degree	Camp
Male	less than 40 years	Bachelor degree	Camp
Male	less than 50 years	Graduate studies	City
Male	less than 30 years	Graduate studies	City
Female	less than 30 years	Diploma	Camp
Female	less than 30 years	Bachelor degree	City
Female	less than 30 years	Bachelor degree	Camp
Female	less than 40 years	Bachelor degree	Village
Female	less than 50 years	Bachelor degree	City
Female	less than 30 years	Graduate studies	Camp
Female	less than 30 years	Bachelor degree	Camp
Male	less than 40 years	Bachelor degree	Village
Female	less than 30 years	Bachelor degree	City
Female	less than 30 years	Diploma	Camp
Male	less than 50 years	Bachelor degree	Village
Male	less than 30 years	Bachelor degree	Village
Male	less than 30 years	Bachelor degree	City
Male	less than 50 years	Graduate studies	City
Female	less than 30 years	Bachelor degree	City
Female	less than 40 years	Bachelor degree	City
Female	less than 30 years	Bachelor degree	Village
Female	less than 30 years	Graduate studies	Camp
Female	less than 30 years	Bachelor degree	Camp
Male	less than 30 years	Graduate studies	City
Male	less than 60 years	Graduate studies	City
Female	less than 30 years	Diploma	City
Male	less than 50 years	Graduate studies	City
Female	less than 50 years	Bachelor degree	City
Female	less than 30 years	Graduate studies	Village
Male	less than 30 years	Diploma	City
Female	less than 30 years	Bachelor degree	City
Female	less than 30 years	Bachelor degree	City
Female	less than 40 years	Graduate studies	City
Female	less than 30 years	Bachelor degree	City
Male	less than 50 years	Graduate studies	City
Male	less than 60 years	Bachelor degree	City
Male	less than 30 years	Bachelor degree	City
Female	less than 60 years	Bachelor degree	Village
Female	less than 40 years	Graduate studies	Village
Male	less than 30 years	Diploma	City
Male	less than 50 years	Bachelor degree	Village
Female	less than 40 years	Bachelor degree	Village
Male	less than 60 years	Diploma	Village
Female	less than 30 years	Bachelor degree	Village
Male	less than 60 years	Bachelor degree	Village
Female	less than 30 years	Bachelor degree	Camp
Male	less than 40 years	Bachelor degree	Village
Female	less than 30 years	Bachelor degree	Village
Female	less than 30 years	Diploma	Camp
Male	less than 30 years	Bachelor degree	City
Female	less than 30 years	Bachelor degree	City
Female	less than 30 years	Graduate studies	Camp
Female	less than 30 years	Bachelor degree	City
Male	less than 40 years	Tawjihi or less	City
Female	less than 30 years	Bachelor degree	City

Female	less than 30 years	Graduate studies	Village
Female	less than 30 years	Bachelor degree	City
Female	less than 30 years	Bachelor degree	City
Female	less than 40 years	Graduate studies	City
Male	less than 50 years	Graduate studies	City
Male	less than 50 years	Bachelor degree	Village
Male	less than 50 years	Graduate studies	Village
Female	less than 30 years	Bachelor degree	City
Male	less than 30 years	Bachelor degree	Camp
Male	less than 30 years	Bachelor degree	Village
Male	less than 40 years	Diploma	Village
Male	less than 60 years	Graduate studies	City
Male	less than 40 years	Bachelor degree	Village
Female	less than 30 years	Bachelor degree	Village
Male	less than 60 years	Bachelor degree	City
Female	less than 40 years	Bachelor degree	City
Male	less than 40 years	Tawjihi or less	Village
Male	less than 30 years	Bachelor degree	Village
Male	less than 40 years	Graduate studies	City
Male	less than 40 years	Graduate studies	Village
Female	less than 30 years	Bachelor degree	Village
Male	less than 40 years	Graduate studies	City
Female	less than 30 years	Bachelor degree	City
Male	less than 50 years	Diploma	Village
Male	less than 60 years	Graduate studies	City
Male	less than 50 years	Bachelor degree	Village
Male	less than 50 years	Bachelor degree	Village
Female	less than 40 years	Bachelor degree	City
Male	less than 60 years	Graduate studies	Village
Female	less than 30 years	Bachelor degree	Village
Female	less than 30 years	Bachelor degree	City
Male	less than 50 years	Bachelor degree	Village
Male	less than 60 years	Graduate studies	City
Male	less than 60 years	Graduate studies	City
Female	less than 50 years	Bachelor degree	City
Male	less than 50 years	Bachelor degree	Village
Male	less than 50 years	Graduate studies	Camp
Male	less than 30 years	Bachelor degree	Village
Female	less than 30 years	Bachelor degree	Village
Male	less than 50 years	Graduate studies	City
Male	less than 30 years	Bachelor degree	Village
Female	less than 30 years	Bachelor degree	Camp
Male	less than 50 years	Bachelor degree	Village
Male	less than 30 years	Bachelor degree	City
Male	less than 40 years	Graduate studies	Village
Female	less than 30 years	Bachelor degree	Village
Female	less than 40 years	Graduate studies	City
Male	less than 50 years	Graduate studies	City
Male	less than 60 years	Bachelor degree	City
Male	less than 60 years	Graduate studies	Camp
Male	less than 40 years	Bachelor degree	Village
Female	less than 30 years	Bachelor degree	Village
Male	less than 40 years	Tawjihi or less	Village
Male	less than 30 years	Bachelor degree	Village
Male	less than 30 years	Bachelor degree	City

Female	less than 30 years	Bachelor degree	Village
Male	less than 30 years	Bachelor degree	Village
Male	less than 40 years	Bachelor degree	City
Male	less than 40 years	Tawjihi or less	City
Female	less than 30 years	Bachelor degree	Village
Male	less than 40 years	Graduate studies	City
Male	less than 50 years	Graduate studies	City
Male	less than 40 years	Bachelor degree	City
Female	less than 60 years	Graduate studies	City
Female	less than 30 years	Bachelor degree	City
Female	less than 30 years	Graduate studies	City
Male	less than 30 years	Graduate studies	City
Male	less than 40 years	Bachelor degree	City
Male	less than 50 years	Graduate studies	Village
Female	less than 40 years	Bachelor degree	Village
Female	less than 40 years	Bachelor degree	Village
Male	less than 40 years	Bachelor degree	Village
Female	less than 30 years	Bachelor degree	City
Male	less than 40 years	Bachelor degree	City
Male	less than 30 years	Bachelor degree	City
Female	less than 40 years	Bachelor degree	Village
Male	less than 50 years	Graduate studies	Camp
Female	less than 30 years	Diploma	Village
Female	less than 30 years	Bachelor degree	Camp
Female	less than 60 years	Tawjihi or less	Village
Male	less than 50 years	Graduate studies	City
Male	less than 50 years	Bachelor degree	Village
Male	less than 50 years	Bachelor degree	City
Female	less than 30 years	Bachelor degree	City
Female	less than 30 years	Bachelor degree	City
Male	less than 40 years	Graduate studies	City
Male	less than 30 years	Graduate studies	Village
Male	less than 30 years	Diploma	City
Female	less than 30 years	Bachelor degree	Village
Female	less than 30 years	Bachelor degree	City
Male	less than 60 years	Graduate studies	City
Male	less than 40 years	Graduate studies	Village
Female	less than 30 years	Bachelor degree	City
Male	less than 30 years	Bachelor degree	Village
Male	less than 40 years	Bachelor degree	City
Male	less than 50 years	Graduate studies	City
Male	less than 50 years	Graduate studies	Village
Male	less than 30 years	Tawjihi or less	Village
Male	less than 30 years	Tawjihi or less	Village
Male	less than 30 years	Bachelor degree	Village
Male	less than 30 years	Tawjihi or less	Village
Female	less than 30 years	Graduate studies	City
Male	less than 30 years	Bachelor degree	City
Female	less than 50 years	Diploma	City
Female	less than 30 years	Bachelor degree	Camp
Male	less than 30 years	Bachelor degree	Village
Male	less than 50 years	Bachelor degree	Village
Male	less than 30 years	Bachelor degree	Village
Male	less than 30 years	Bachelor degree	City
Female	less than 30 years	Bachelor degree	City

Male	less than 50 years	Bachelor degree	City
Male	less than 30 years	Graduate studies	Village
Female	less than 30 years	Bachelor degree	Camp
Male	less than 40 years	Bachelor degree	Village
Male	less than 60 years	Bachelor degree	Village
Female	less than 30 years	Bachelor degree	City
Male	less than 40 years	Tawjihi or less	City
Male	less than 40 years	Graduate studies	Camp
Female	less than 30 years	Bachelor degree	Camp
Male	less than 30 years	Bachelor degree	Village
Male	less than 60 years	Bachelor degree	Village
Male	less than 40 years	Graduate studies	Village
Female	less than 40 years	Diploma	Village
Female	less than 30 years	Bachelor degree	City
Female	less than 30 years	Graduate studies	Village
Male	less than 40 years	Diploma	Village
Male	less than 30 years	Graduate studies	Village
Female	less than 40 years	Bachelor degree	Village
Female	less than 30 years	Tawjihi or less	Village
Female	less than 40 years	Bachelor degree	Village
Female	less than 40 years	Bachelor degree	Village
Female	less than 40 years	Bachelor degree	Village
Male	less than 50 years	Bachelor degree	City
Female	less than 30 years	Graduate studies	Village
Female	less than 30 years	Graduate studies	City
Female	less than 60 years	Diploma	Village
Female	less than 30 years	Graduate studies	City
Female	less than 50 years	Bachelor degree	Village
Female	less than 40 years	Bachelor degree	City
Male	less than 60 years	Diploma	City
Female	less than 30 years	Bachelor degree	City
Male	less than 40 years	Graduate studies	Village
Male	less than 30 years	Bachelor degree	Village
Female	less than 50 years	Tawjihi or less	City
Female	less than 30 years	Bachelor degree	City
Female	less than 50 years	Bachelor degree	Village
Female	less than 40 years	Bachelor degree	Village
Male	less than 30 years	Bachelor degree	Village
Female	less than 40 years	Bachelor degree	Village
Male	less than 50 years	Bachelor degree	Village
Male	less than 30 years	Bachelor degree	City
Male	less than 40 years	Graduate studies	City
Male	less than 50 years	Graduate studies	City
Female	less than 30 years	Bachelor degree	Village
Female	less than 40 years	Bachelor degree	City
Female	less than 30 years	Bachelor degree	City
Female	less than 30 years	Bachelor degree	Village
Female	less than 50 years	Tawjihi or less	Village
Male	less than 50 years	Graduate studies	City
Male	less than 50 years	Tawjihi or less	Village
Male	less than 60 years	Graduate studies	Village
Female	less than 30 years	Bachelor degree	City
Female	less than 40 years	Bachelor degree	Village
Female	less than 30 years	Bachelor degree	Village
Female	less than 30 years	Bachelor degree	City

Female	less than 30 years	Bachelor degree	Village
Male	less than 40 years	Diploma	Village
Female	less than 30 years	Bachelor degree	City
Female	less than 30 years	Bachelor degree	Village
Male	less than 40 years	Bachelor degree	Village
Male	less than 50 years	Graduate studies	City
Male	less than 50 years	Bachelor degree	Village
Female	less than 30 years	Bachelor degree	City
Male	less than 30 years	Bachelor degree	Village
Male	less than 60 years	Tawjihi or less	Village
Male	less than 30 years	Tawjihi or less	City
Male	less than 30 years	Bachelor degree	City
Male	less than 60 years	Graduate studies	Camp
Male	less than 30 years	Bachelor degree	Village
Female	less than 40 years	Bachelor degree	Camp
Male	less than 40 years	Bachelor degree	Village
Male	less than 50 years	Diploma	Village
Male	less than 40 years	Bachelor degree	Village
Female	less than 30 years	Bachelor degree	Village
Male	less than 60 years	Graduate studies	City
Female	less than 30 years	Bachelor degree	City
Male	less than 50 years	Tawjihi or less	Village
Female	less than 40 years	Bachelor degree	Village
Female	less than 30 years	Tawjihi or less	City
Male	less than 50 years	Bachelor degree	City
Male	less than 30 years	Bachelor degree	City
Female	less than 30 years	Bachelor degree	City
Male	less than 30 years	Tawjihi or less	Village
Male	less than 30 years	Tawjihi or less	Village
Male	less than 30 years	Tawjihi or less	Village
Male	less than 30 years	Diploma	Village
Male	less than 30 years	Bachelor degree	Village
Female	less than 30 years	Bachelor degree	City
Female	less than 30 years	Bachelor degree	City
Male	less than 50 years	Graduate studies	City
Male	less than 30 years	Bachelor degree	City
Female	less than 60 years	Graduate studies	City
Male	less than 60 years	Diploma	City
Male	less than 30 years	Bachelor degree	City
Female	less than 30 years	Graduate studies	Village
Male	less than 50 years	Graduate studies	Village
Female	less than 60 years	Bachelor degree	City
Male	less than 30 years	Bachelor degree	Village
Female	less than 30 years	Graduate studies	Village
Female	less than 30 years	Bachelor degree	City
Male	less than 40 years	Bachelor degree	Village
Female	less than 50 years	Graduate studies	City
Male	less than 40 years	Graduate studies	City
Female	less than 30 years	Bachelor degree	Village
Female	less than 30 years	Bachelor degree	Village
Male	less than 50 years	Graduate studies	City
Female	less than 30 years	Diploma	Camp
Male	less than 50 years	Diploma	Camp
Female	less than 30 years	Bachelor degree	Village
Female	less than 30 years	Graduate studies	City

Female	less than 50 years	Graduate studies	City
Male	less than 50 years	Graduate studies	City
Female	less than 40 years	Graduate studies	Village
Male	less than 50 years	Bachelor degree	Village
Female	less than 50 years	Bachelor degree	Village
Female	less than 50 years	Bachelor degree	City
Male	less than 40 years	Graduate studies	City
Male	less than 30 years	Bachelor degree	City
Male	less than 40 years	Bachelor degree	Village
Female	less than 30 years	Bachelor degree	Village
Female	less than 30 years	Bachelor degree	City
Female	less than 30 years	Bachelor degree	Village
Female	less than 30 years	Bachelor degree	City
Male	less than 40 years	Bachelor degree	Village
Female	less than 30 years	Bachelor degree	Village
Male	less than 60 years	Graduate studies	Village
Female	less than 30 years	Graduate studies	City

One of the reasons for viol	violence, physical or	One of the reasons for viol	One of the reasons for viol
Agree	Agree	Agree	Strongly agree
Disagree	Agree	Disagree	Agree
I do not know	Agree	Agree	Agree
I do not know	Agree	Agree	Agree
Strongly disagree	Strongly disagree	Disagree	I do not know
Disagree	Agree	Strongly agree	Agree
Disagree	Strongly agree	Agree	Agree
I do not know	Agree	Strongly agree	Agree
Strongly agree	Agree	Agree	Agree
I do not know	Agree	Strongly agree	Strongly agree
Agree	Agree	Agree	Strongly agree
Strongly disagree	Disagree	Agree	Strongly disagree
Disagree	Agree	Agree	Disagree
Agree	Strongly agree	Agree	Agree
Strongly agree	Strongly agree	Strongly agree	Strongly agree
Agree	Agree	Agree	Strongly agree
I do not know	Agree	Agree	Agree
Agree	Agree	Agree	Agree
Strongly agree	Strongly agree	Strongly agree	Strongly agree
Agree	Agree	Strongly disagree	Disagree
Agree	Strongly agree	Agree	Agree
I do not know	Strongly disagree	Strongly agree	Strongly agree
Agree	Agree	Disagree	Agree
Disagree	Disagree	Agree	Disagree
Disagree	Disagree	Agree	Disagree
Strongly agree	Strongly agree	Agree	Strongly agree
Agree	Disagree	Disagree	Agree
Disagree	Disagree	I do not know	Disagree
Agree	Agree	Strongly agree	Disagree
Strongly agree	Agree	Strongly disagree	Disagree
Disagree	Disagree	Disagree	Agree
I do not know	Agree	Agree	I do not know
Agree	Agree	I do not know	Strongly agree
Strongly agree	Agree	Agree	Strongly agree
Disagree	Disagree	Disagree	Agree
Strongly disagree	Strongly disagree	Strongly disagree	Strongly disagree
Agree	Agree	Agree	Agree
Disagree	Disagree	Disagree	Agree
Agree	Agree	Agree	Agree
Agree	Agree	Agree	Strongly agree
Agree	Agree	Strongly agree	Strongly agree
Strongly agree	Strongly agree	Strongly agree	Strongly agree
Agree	Strongly agree	Strongly agree	Strongly agree
Agree	Strongly agree	Disagree	Agree
Strongly agree	Strongly agree	Strongly agree	Strongly agree
Strongly agree	Strongly agree	Strongly agree	Agree
Agree	Strongly agree	Strongly agree	Agree
Disagree	Disagree	Agree	Strongly agree

Agree	Strongly disagree	Disagree	Agree
Agree	Disagree	Agree	Agree
I do not know	Agree	Agree	Strongly agree
Strongly disagree	Strongly disagree	Agree	Agree
Disagree	Agree	Agree	Agree
Agree	Agree	Agree	Disagree
Agree	Agree	Disagree	Agree
Agree	Agree	Agree	Agree
Agree	I do not know	Agree	Agree
I do not know	I do not know	Disagree	Disagree
Agree	Strongly agree	Agree	Strongly agree
Strongly agree	Agree	Strongly agree	Strongly agree
Agree	Strongly agree	I do not know	Strongly agree
Agree	Agree	I do not know	Agree
Strongly agree	Strongly agree	Strongly agree	Strongly agree
I do not know	Agree	Agree	Agree
Agree	Agree	Agree	Agree
Strongly disagree	Strongly disagree	Disagree	Agree
Agree	Agree	Agree	Strongly agree
Strongly agree	Agree	Agree	Agree
Disagree	Agree	Disagree	Agree
Agree	Strongly agree	Strongly agree	Agree
Disagree	Disagree	I do not know	Agree
Agree	Agree	Agree	Strongly agree
I do not know	Strongly agree	Agree	Strongly agree
Disagree	Agree	Agree	Strongly agree
Disagree	Disagree	Strongly disagree	I do not know
Agree	Agree	Agree	Agree
Agree	Agree	Agree	Agree
Disagree	I do not know	Disagree	Agree
Agree	Agree	Agree	Agree
Strongly disagree	Agree	Agree	Agree
Disagree	Disagree	Disagree	Agree
I do not know	Agree	Agree	Agree
I do not know	Agree	I do not know	Agree
Agree	Strongly agree	Agree	Agree
Agree	Agree	Strongly agree	Strongly agree
Strongly disagree	Strongly disagree	Disagree	Strongly disagree
I do not know	I do not know	Agree	Agree
Agree	Disagree	Agree	Agree
Strongly agree	Strongly agree	Strongly agree	Strongly agree
Disagree	Disagree	Disagree	Disagree
Agree	Strongly disagree	I do not know	I do not know
Agree	Agree	Agree	I do not know
Disagree	Agree	Agree	Agree
I do not know	Strongly agree	Strongly agree	Strongly agree
Strongly agree	Strongly agree	Strongly agree	Strongly agree
Strongly agree	Strongly agree	Strongly agree	Strongly agree
I do not know	I do not know	I do not know	Agree
Disagree	Agree	Strongly agree	Agree
Agree	Strongly agree	Agree	Agree
Strongly agree	Strongly agree	Strongly agree	Strongly agree
Agree	Agree	Strongly agree	Agree
Strongly agree	Strongly agree	Agree	Strongly agree
Agree	Agree	Agree	Agree

I do not know	Agree	Disagree	Disagree
Strongly disagree	Strongly disagree	Disagree	I do not know
Agree	I do not know	Agree	I do not know
Agree	Strongly agree	Agree	Strongly agree
Strongly disagree	Disagree	Disagree	I do not know
Strongly agree	Agree	Agree	Agree
Agree	Agree	Agree	Agree
I do not know	Agree	I do not know	Disagree
Agree	Agree	Agree	Strongly agree
Agree	Strongly agree	Agree	Agree
Strongly agree	Strongly agree	Strongly agree	Agree
Agree	Agree	Agree	Disagree
Disagree	Agree	Agree	Agree
Disagree	Disagree	Disagree	Disagree
I do not know	Agree	I do not know	Agree
Disagree	Strongly agree	Strongly agree	Agree
Agree	Strongly agree	Disagree	Strongly agree
Agree	I do not know	Disagree	Agree
Strongly disagree	Agree	Agree	Agree
Agree	Agree	Agree	Agree
Agree	Agree	Agree	Agree
Agree	I do not know	Agree	Agree
Agree	Agree	I do not know	Strongly agree
I do not know	Agree	Agree	Strongly agree
Agree	Strongly agree	Strongly agree	Strongly agree
Agree	Strongly agree	Agree	Agree
I do not know	Strongly agree	Strongly agree	Strongly agree
Agree	I do not know	Agree	Agree
Agree	Agree	Agree	Disagree
Agree	Agree	Agree	Agree
Strongly disagree	Agree	Agree	I do not know
Strongly agree	Strongly agree	Strongly agree	Strongly agree
Agree	Strongly agree	Strongly agree	Strongly agree
Strongly disagree	Strongly disagree	Strongly disagree	Strongly disagree
Disagree	Agree	Agree	Disagree
Agree	Agree	Strongly agree	Strongly agree
Strongly agree	Agree	Agree	Agree
Strongly agree	Agree	Agree	Agree
Disagree	Disagree	Disagree	Agree
Agree	Agree	Disagree	Agree
Disagree	Disagree	Agree	Agree
Agree	I do not know	Agree	Agree
Agree	Agree	Strongly agree	Agree
Agree	Agree	Agree	Agree
Agree	Agree	Agree	Agree
Agree	Disagree	Agree	Agree
Agree	Strongly agree	Agree	Strongly agree
Disagree	Disagree	Disagree	Strongly disagree
Agree	Strongly agree	Agree	I do not know
Disagree	Agree	Agree	Agree
Agree	Agree	Agree	Strongly agree
Strongly agree	Agree	Strongly agree	Strongly agree
Agree	Agree	Agree	Agree
Strongly disagree	Agree	Strongly disagree	Strongly disagree
Strongly agree	Strongly agree	Agree	Agree

Agree	Agree	Strongly agree	Strongly agree
Agree	Agree	Agree	Agree
Agree	Strongly agree	Strongly agree	Strongly agree
Strongly agree	Strongly agree	Strongly agree	Strongly agree
Strongly agree	Agree	Strongly agree	Disagree
I do not know	Agree	Agree	Agree
Agree	Agree	Disagree	Agree
Agree	Strongly agree	Agree	Strongly agree
Strongly agree	Strongly agree	Agree	Strongly agree
Disagree	Strongly disagree	Disagree	Agree
Strongly agree	Strongly agree	Strongly agree	Strongly agree
Disagree	Strongly agree	I do not know	I do not know
Agree	Agree	Agree	Agree
Agree	Agree	Agree	Strongly agree
Agree	Agree	Agree	Agree
I do not know	Agree	Agree	Agree
Strongly agree	Strongly agree	Strongly agree	Strongly agree
Disagree	Agree	Agree	Agree
Disagree	Disagree	Disagree	Disagree
Agree	Agree	Agree	Agree
Agree	Agree	Agree	I do not know
Strongly agree	Strongly agree	Strongly agree	Strongly agree
Agree	Strongly agree	Agree	Strongly agree
Strongly agree	Strongly agree	Agree	Agree
Agree	Agree	Disagree	Agree
I do not know	Agree	Agree	Strongly agree
Strongly agree	Agree	Disagree	Agree
Strongly disagree	Strongly disagree	Strongly disagree	Strongly disagree
Agree	Agree	I do not know	Strongly agree
Strongly disagree	I do not know	Agree	Agree
Disagree	Agree	Agree	Strongly agree
Strongly agree	Strongly agree	Agree	I do not know
Disagree	Disagree	Agree	Agree
Strongly agree	Agree	Agree	Disagree
Agree	Agree	Agree	Agree
Agree	Strongly agree	Strongly agree	I do not know
Agree	Agree	Agree	Agree
Agree	Agree	Agree	Strongly agree
Agree	Agree	Agree	Agree
Agree	Agree	Agree	Agree
Agree	Agree	Agree	Strongly agree
Strongly agree	Agree	Agree	Strongly agree
Agree	Agree	Agree	Agree
Agree	Agree	Agree	Strongly agree
Disagree	Disagree	Agree	Agree
Disagree	Agree	Disagree	Agree
Agree	Agree	Agree	Strongly agree
Strongly agree	Agree	Strongly agree	Agree
Strongly agree	Strongly agree	Strongly agree	Strongly agree
Agree	Agree	Agree	I do not know
Agree	Agree	Agree	Agree
Agree	Agree	Agree	Strongly agree
Agree	I do not know	Strongly disagree	Strongly disagree
Agree	Agree	Agree	Strongly agree
Strongly disagree	Agree	Agree	Agree

I do not know	Agree	I do not know	Agree
Strongly agree	Strongly agree	Strongly agree	Strongly agree
Agree	Agree	Agree	I do not know
Agree	Agree	Agree	Strongly agree
Agree	Agree	Strongly agree	Strongly agree
I do not know	I do not know	Agree	I do not know
Agree	Agree	Agree	Agree
Agree	Agree	Agree	Strongly agree
Agree	Agree	Strongly agree	I do not know
Disagree	Agree	Disagree	Agree
Agree	Agree	Agree	Strongly agree
Agree	Disagree	Agree	Disagree
Agree	Agree	Strongly agree	Strongly agree
Agree	I do not know	Disagree	Disagree
Agree	Disagree	Agree	Disagree
Strongly disagree	Disagree	Disagree	Agree
Disagree	Disagree	Disagree	Agree
Strongly agree	Agree	Agree	Agree
Agree	Agree	Strongly agree	Agree
Agree	Strongly agree	Strongly agree	Agree
Agree	Strongly agree	I do not know	Strongly agree
Agree	Strongly agree	Agree	Agree
I do not know	Agree	Agree	Strongly agree
Strongly agree	Strongly agree	Strongly agree	Strongly agree
Strongly agree	I do not know	Strongly agree	Strongly agree
Strongly disagree	Strongly disagree	Disagree	Disagree
Strongly disagree	Strongly disagree	Agree	Agree
Strongly agree	Strongly agree	Strongly agree	Strongly agree
Disagree	I do not know	Disagree	Strongly agree
Agree	Strongly agree	Strongly agree	Disagree
Agree	Agree	Agree	Strongly agree
Disagree	Agree	Agree	Disagree
I do not know	Agree	Agree	Agree
I do not know	Agree	Agree	Agree
Agree	Agree	Agree	Strongly agree
Strongly disagree	Strongly disagree	Agree	Agree
Strongly agree	Strongly agree	Agree	Agree
I do not know	I do not know	I do not know	Disagree
Agree	Agree	Agree	Agree
Strongly agree	Agree	Agree	Agree
Agree	Agree	Agree	Agree
Agree	Agree	Agree	Agree
Strongly agree	Strongly agree	Strongly agree	Agree
Disagree	Disagree	Disagree	Disagree
Disagree	Disagree	Disagree	Disagree
Disagree	Agree	I do not know	Agree
Disagree	Disagree	Disagree	Agree
Agree	Strongly agree	Strongly agree	Strongly agree
Agree	Agree	I do not know	Agree
I do not know	I do not know	Agree	Agree
I do not know	Agree	I do not know	Agree
Agree	Strongly agree	Agree	Strongly agree
I do not know	Strongly agree	I do not know	Agree
Agree	Strongly agree	Agree	Strongly agree
Agree	Agree	Disagree	Disagree

Strongly agree	Strongly agree	Strongly agree	Strongly agree
Disagree	I do not know	Strongly agree	Agree
Agree	Agree	Agree	Agree
Agree	Agree	Agree	Agree
I do not know	Strongly agree	Disagree	I do not know
Agree	Agree	Agree	Agree
Disagree	Disagree	Disagree	Disagree
Disagree	Agree	Agree	Agree
Agree	Strongly agree	Strongly agree	Strongly agree
Agree	Strongly agree	Agree	Agree
I do not know	Strongly agree	Agree	Agree
Agree	Strongly agree	Strongly agree	Strongly agree
Disagree	Disagree	Agree	Agree
Disagree	Strongly agree	Agree	Agree
Agree	Disagree	Agree	Agree
Agree	Agree	Agree	Agree
Agree	Agree	Agree	Strongly agree
Agree	Strongly agree	Agree	Agree
Disagree	Disagree	Disagree	Agree
Strongly disagree	Disagree	Agree	Disagree
Disagree	Disagree	Agree	Disagree
Strongly agree	Strongly agree	Strongly agree	Strongly agree
I do not know	I do not know	Disagree	Disagree
Strongly agree	Agree	Agree	Strongly agree
Disagree	Agree	I do not know	Disagree
Agree	Agree	Agree	Agree
Strongly agree	Strongly agree	Strongly agree	Strongly agree
Disagree	Disagree	Disagree	Agree
I do not know	Agree	Agree	Strongly agree
I do not know	Disagree	Agree	Agree
Disagree	Agree	Agree	Agree
Agree	Disagree	Disagree	Agree
I do not know	Agree	Agree	I do not know
Agree	Agree	Agree	Agree
Strongly disagree	Disagree	Disagree	Strongly disagree
Agree	Agree	I do not know	Agree
Agree	I do not know	Agree	Agree
Agree	Agree	I do not know	Agree
Agree	Agree	Agree	Agree
Agree	Agree	I do not know	Agree
Agree	Agree	Agree	Disagree
Disagree	Agree	Strongly agree	Strongly agree
Agree	Agree	Strongly agree	Strongly agree
Disagree	Disagree	Agree	Disagree
Agree	Agree	Agree	Agree
Disagree	Agree	Agree	Strongly agree
Agree	Strongly agree	Agree	Agree
Agree	Agree	Agree	Agree
Agree	Agree	I do not know	Agree
Agree	Agree	Agree	Agree
Disagree	Agree	Agree	Strongly agree
Strongly agree	Strongly agree	Strongly agree	Agree
Strongly agree	Agree	Agree	Strongly agree
Disagree	Agree	Agree	Agree
Agree	Strongly agree	Strongly agree	Agree

Agree	I do not know	Agree	Agree
Strongly agree	Strongly agree	Strongly agree	Strongly agree
I do not know	I do not know	I do not know	Agree
Agree	Strongly agree	Agree	Strongly agree
Strongly agree	Strongly agree	Strongly agree	Agree
Strongly agree	Strongly agree	Strongly agree	Agree
Agree	Agree	Agree	Disagree
Disagree	Agree	Agree	Agree
I do not know	Agree	Agree	I do not know
Strongly agree	Strongly agree	Agree	Agree
Agree	Agree	Agree	Agree
Disagree	Agree	Disagree	Agree
Strongly disagree	Strongly disagree	Strongly disagree	Strongly disagree
Agree	Agree	Strongly agree	Agree
Disagree	Agree	Agree	Disagree
Disagree	Disagree	Disagree	Agree
Strongly disagree	I do not know	Agree	Agree
Agree	Agree	Strongly agree	Agree
Disagree	I do not know	I do not know	Agree
Agree	Strongly agree	Agree	Agree
Strongly disagree	Disagree	Strongly disagree	Disagree
Agree	Agree	Agree	Strongly agree
Agree	Strongly agree	Agree	Strongly disagree
Disagree	Disagree	Strongly disagree	Agree
I do not know	Strongly agree	Agree	Agree
Strongly agree	Agree	Agree	Agree
Agree	Strongly agree	Strongly agree	Strongly agree
Agree	Agree	Agree	Agree
Strongly agree	Strongly disagree	Strongly agree	Strongly agree
Agree	Strongly agree	Strongly agree	Strongly agree
Agree	Agree	Strongly agree	Agree
Agree	Agree	Agree	Agree
Agree	Agree	Agree	Strongly agree
Agree	Agree	Agree	Agree
Strongly agree	Strongly agree	Strongly agree	Strongly agree
Agree	Strongly agree	Strongly agree	Strongly agree
Strongly agree	Strongly agree	Strongly agree	Strongly agree
I do not know	Agree	Strongly disagree	Strongly disagree
Disagree	Disagree	Disagree	Disagree
Strongly agree	Strongly agree	Strongly agree	Strongly agree
Strongly agree	Strongly agree	Strongly agree	Strongly agree
Strongly agree	Agree	Strongly disagree	Agree
Disagree	Disagree	Disagree	Agree
Disagree	I do not know	Agree	I do not know
Agree	Agree	Agree	Agree
Agree	Agree	Agree	I do not know
Disagree	Strongly agree	I do not know	Agree
Strongly agree	Agree	Strongly agree	Strongly agree
Agree	Agree	Agree	Strongly agree
Strongly agree	Strongly agree	Strongly agree	Strongly agree
Disagree	Disagree	Disagree	Agree
Agree	Agree	Agree	Strongly agree
Agree	Agree	Strongly agree	Strongly agree
Agree	I do not know	Disagree	Agree
Disagree	I do not know	Disagree	Disagree

Agree	Agree	Agree	Strongly agree
Agree	Agree	Agree	Agree
Disagree	Disagree	I do not know	I do not know
Disagree	Strongly disagree	Agree	Agree
Agree	Agree	Agree	Strongly agree
I do not know	Strongly agree	Agree	Agree
Agree	Agree	Disagree	Agree
Agree	Strongly agree	Agree	Strongly agree
Disagree	Disagree	I do not know	Agree
I do not know	I do not know	I do not know	I do not know
Disagree	Disagree	Agree	Strongly agree
Agree	Agree	Strongly agree	Agree
Strongly disagree	Agree	Agree	Strongly agree
Agree	Agree	Agree	Strongly agree
Strongly agree	Strongly agree	Strongly agree	Agree
Strongly agree	Agree	Strongly agree	Agree
Disagree	Agree	Disagree	Agree
Disagree	Agree	I do not know	Disagree
Disagree	Disagree	Disagree	Agree
Agree	Agree	Agree	I do not know
I do not know	Strongly disagree	Agree	Strongly agree
Agree	Agree	Strongly agree	Strongly agree
Strongly agree	Agree	Agree	Agree
Disagree	Agree	Disagree	Agree
Agree	Agree	I do not know	Strongly disagree
Agree	Agree	Disagree	Agree
Agree	Agree	I do not know	Agree
Agree	Agree	Agree	Agree
Strongly agree	Agree	Agree	Agree
Agree	I do not know	Agree	Strongly agree
Agree	Strongly agree	Agree	Strongly agree
Agree	Agree	Agree	Agree
Strongly agree	Strongly agree	Strongly agree	Strongly agree
Strongly agree	Strongly agree	Strongly agree	Strongly agree
Agree	Agree	Strongly agree	Strongly agree
I do not know	Agree	Agree	Agree
Agree	Agree	Agree	Strongly agree
Strongly agree	Strongly agree	Strongly agree	Strongly agree
Agree	Agree	Agree	Agree
Strongly disagree	Strongly disagree	Strongly disagree	Strongly disagree
Strongly agree	Agree	Agree	Agree
I do not know	Agree	Agree	Agree
Disagree	Disagree	Disagree	Disagree
Agree	Agree	Agree	Agree
Agree	Agree	Agree	Agree
Agree	Strongly agree	Agree	Agree
Agree	Agree	Strongly agree	Strongly agree
Agree	Agree	Agree	Agree
Agree	Agree	Strongly agree	Agree
Strongly agree	Agree	Agree	Strongly agree
Agree	Agree	Agree	Agree
Agree	Strongly agree	Strongly agree	Strongly agree
Agree	Agree	Strongly agree	Agree
Agree	Agree	Strongly agree	Strongly agree
Agree	Agree	I do not know	Strongly agree

Agree	Agree	Strongly disagree	Strongly disagree
Agree	Strongly agree	Strongly agree	Strongly agree
Agree	Agree	I do not know	Agree
Disagree	Disagree	I do not know	Agree
Strongly disagree	Strongly disagree	Disagree	Disagree
Strongly disagree	Agree	Disagree	Agree
Agree	Agree	I do not know	Disagree
I do not know	Agree	Agree	Disagree
Agree	Agree	Agree	Strongly agree
Agree	Agree	I do not know	Disagree
Agree	Disagree	Strongly agree	Agree
Disagree	Strongly agree	Disagree	Strongly agree
Agree	Agree	Agree	I do not know
Agree	Agree	Agree	Strongly agree
Agree	Agree	Agree	Agree
Strongly disagree	Agree	I do not know	Agree
Agree	Agree	Agree	Agree
Agree	Agree	Strongly agree	Agree
Agree	Strongly agree	Agree	Strongly agree
I do not know	Agree	Agree	Agree
Agree	Strongly agree	Agree	Strongly agree
Strongly disagree	Agree	Agree	Strongly disagree
Strongly agree	Agree	Agree	Strongly agree
Agree	Agree	Agree	Agree
Agree	Agree	Disagree	Disagree
Disagree	Disagree	Strongly disagree	Disagree
Disagree	Disagree	Disagree	I do not know
Agree	Strongly agree	Strongly agree	Strongly agree
Strongly agree	Strongly agree	Strongly agree	Strongly agree
Strongly agree	Agree	Strongly disagree	Strongly agree
Agree	Strongly agree	Agree	Agree
Disagree	Agree	Agree	Agree
Disagree	Agree	Agree	Disagree
I do not know	Agree	Agree	Agree
Agree	Agree	Agree	Agree
I do not know	Agree	Agree	Agree
I do not know	Agree	Agree	Agree
Agree	Agree	Agree	Disagree
Agree	Agree	Agree	Agree
Disagree	Disagree	Agree	Agree
Disagree	Agree	Agree	Agree
Agree	Agree	Agree	Agree
Agree	Agree	Agree	I do not know
Strongly agree	Strongly agree	Strongly agree	Strongly agree
Agree	Agree	Strongly agree	Agree
Agree	Agree	Agree	Agree
I do not know	Agree	Agree	Agree
Agree	Agree	I do not know	Agree
Agree	Agree	Strongly agree	Agree
Disagree	Agree	Agree	Strongly agree
Agree	Agree	Agree	Agree
Strongly agree	Strongly disagree	Strongly disagree	Strongly disagree
Strongly agree	Agree	Strongly agree	Strongly agree
Strongly agree	Strongly agree	Strongly agree	Strongly agree
Agree	Agree	Agree	Strongly disagree

Agree	Agree	Agree	Strongly agree
Disagree	Disagree	Disagree	Disagree
Strongly agree	Strongly agree	Agree	Strongly agree
Strongly agree	I do not know	Agree	Agree
I do not know	Agree	Agree	Disagree
Agree	Agree	Agree	Strongly agree
Disagree	Agree	Disagree	Disagree
Agree	Agree	Agree	Agree
Agree	Agree	Agree	Agree
Strongly disagree	Strongly disagree	Strongly disagree	Agree
Disagree	Disagree	Disagree	I do not know
Agree	Strongly agree	Strongly agree	Agree
Strongly disagree	Disagree	Disagree	Agree
Disagree	Disagree	Strongly agree	Strongly agree
Disagree	Agree	Agree	Agree
I do not know	Agree	Agree	Agree
Agree	Agree	Strongly agree	Agree

violence, physical or	One of the reasons for viol	One of the reasons for viol	One of the reasons for viol
I do not know	Agree	I do not know	Agree
I do not know	Disagree	Agree	Agree
Strongly agree	Agree	Strongly agree	Strongly agree
Disagree	Strongly agree	Agree	I do not know
Disagree	Disagree	Disagree	Disagree
I do not know	Agree	Agree	Strongly agree
Disagree	Disagree	Disagree	Disagree
Disagree	Strongly agree	Disagree	Agree
Agree	Agree	Agree	Agree
Agree	Strongly agree	Agree	Agree
I do not know	Strongly agree	Strongly agree	Agree
Disagree	Strongly disagree	Strongly disagree	Strongly disagree
Disagree	Strongly agree	Agree	Agree
Strongly agree	Agree	Strongly agree	Strongly agree
Strongly agree	Strongly agree	Strongly agree	Strongly agree
Disagree	Agree	Agree	Agree
I do not know	Agree	Agree	I do not know
Strongly agree	Disagree	Strongly agree	Disagree
Strongly agree	Strongly agree	Strongly agree	Strongly agree
I do not know	Agree	Strongly agree	Strongly agree
Agree	Agree	Strongly agree	Agree
Strongly agree	I do not know	Strongly agree	Agree
Agree	Agree	Disagree	Strongly agree
Agree	Agree	Strongly agree	Agree
Agree	Agree	Strongly agree	Agree
I do not know	Disagree	Agree	Agree
Disagree	Disagree	Strongly disagree	Disagree
Disagree	I do not know	I do not know	Disagree
Strongly disagree	Agree	Agree	I do not know
I do not know	Strongly disagree	Disagree	Strongly disagree
Disagree	Agree	Agree	Agree
Disagree	Agree	I do not know	Disagree
I do not know	I do not know	Agree	Strongly agree
Agree	Strongly agree	Strongly agree	Agree
Disagree	Agree	Strongly agree	Agree
Strongly disagree	Strongly disagree	Strongly disagree	Strongly disagree
I do not know	Agree	I do not know	Strongly agree
Disagree	Disagree	Disagree	Disagree
Agree	Agree	Strongly agree	Strongly agree
Disagree	Agree	Strongly agree	Strongly agree
Agree	Strongly agree	Agree	Agree
Agree	Strongly agree	Strongly agree	Strongly agree
Strongly agree	Strongly agree	Strongly agree	Strongly agree
I do not know	Agree	Strongly agree	Agree
Disagree	Agree	Strongly agree	Strongly agree
Agree	Strongly agree	Strongly agree	Agree
Disagree	Strongly agree	Strongly agree	Strongly agree
I do not know	Strongly agree	Strongly agree	Strongly agree

Disagree	Strongly agree	Strongly agree	Strongly agree
Disagree	Strongly agree	Strongly disagree	Disagree
Agree	Agree	Agree	Agree
Agree	Agree	Agree	Strongly disagree
Agree	Agree	Agree	Agree
Agree	Agree	Strongly agree	Agree
Disagree	Disagree	Strongly agree	Strongly agree
I do not know	Agree	Agree	Agree
Agree	Disagree	Agree	Strongly agree
Agree	Agree	Disagree	Agree
Disagree	Agree	Agree	Agree
Strongly agree	Strongly agree	Strongly agree	Strongly agree
Agree	Agree	I do not know	Agree
Strongly disagree	Strongly disagree	Agree	Agree
I do not know	Agree	I do not know	Agree
Agree	Agree	Agree	Agree
I do not know	Agree	Agree	Agree
Strongly disagree	Strongly disagree	Strongly agree	Strongly agree
Strongly disagree	Disagree	Agree	Agree
Agree	Agree	Agree	Agree
Disagree	I do not know	Disagree	I do not know
Agree	Strongly agree	Strongly agree	Agree
Disagree	Agree	Agree	Disagree
Agree	Strongly agree	Agree	Agree
I do not know	Agree	Agree	Strongly agree
I do not know	Strongly agree	Strongly agree	Strongly agree
Disagree	Agree	Strongly disagree	Disagree
I do not know	Agree	Agree	Agree
Agree	Agree	Agree	Agree
I do not know	I do not know	Agree	Agree
I do not know	Agree	Agree	Agree
Agree	Disagree	Disagree	Disagree
Agree	Agree	Agree	Agree
Agree	Disagree	Agree	Agree
Agree	Agree	Agree	Agree
Agree	Strongly agree	Strongly agree	Agree
Disagree	Agree	Agree	Agree
Disagree	Disagree	Strongly disagree	Disagree
Agree	Agree	Agree	Agree
Agree	Disagree	Agree	Disagree
Agree	Agree	Strongly agree	Strongly agree
Disagree	Strongly disagree	Disagree	Disagree
Strongly disagree	Strongly agree	Strongly agree	Agree
I do not know	Agree	I do not know	I do not know
Agree	Agree	Agree	Agree
Agree	Agree	Agree	Agree
Disagree	Strongly agree	Agree	Agree
Strongly agree	Strongly agree	Strongly agree	Strongly agree
Agree	Agree	Agree	Agree
Agree	Agree	Strongly agree	Agree
Agree	Agree	Agree	Agree
Agree	Strongly agree	Strongly agree	Strongly agree
Disagree	I do not know	Disagree	Agree
Strongly agree	Agree	Strongly agree	Strongly agree
Agree	Disagree	Strongly disagree	I do not know

I do not know	I do not know	Disagree	Strongly disagree
Strongly disagree	I do not know	Strongly disagree	Strongly disagree
Disagree	Disagree	Agree	I do not know
Agree	Agree	Agree	Agree
I do not know	I do not know	I do not know	I do not know
Strongly agree	Strongly agree	Agree	Strongly agree
Agree	Strongly agree	Agree	Agree
Strongly agree	Agree	Disagree	Disagree
Strongly agree	Strongly agree	Strongly agree	Strongly agree
I do not know	Strongly agree	Strongly agree	Strongly agree
I do not know	Agree	Agree	Agree
I do not know	Strongly agree	Strongly agree	Strongly agree
Agree	Agree	Agree	Agree
Disagree	Disagree	Agree	Agree
I do not know	I do not know	Agree	I do not know
Agree	Disagree	Agree	Disagree
I do not know	Disagree	Strongly agree	Agree
Agree	I do not know	Agree	Agree
Agree	Agree	Agree	Agree
I do not know	Agree	Agree	Agree
Agree	Agree	Agree	Agree
I do not know	Agree	I do not know	I do not know
Disagree	Agree	Agree	Agree
Agree	Agree	Disagree	Disagree
Agree	Strongly agree	Strongly agree	Strongly agree
Strongly agree	Agree	Strongly agree	Agree
Strongly agree	Strongly agree	Strongly disagree	Disagree
Strongly agree	Agree	I do not know	Agree
I do not know	Strongly agree	I do not know	Disagree
Agree	Agree	Agree	Agree
Disagree	I do not know	Strongly disagree	Disagree
Strongly agree	Strongly agree	Agree	Strongly agree
Agree	Agree	Disagree	Agree
Strongly disagree	Strongly disagree	Strongly disagree	Strongly disagree
Disagree	Agree	Disagree	Disagree
Strongly agree	Agree	Strongly agree	Agree
Disagree	I do not know	Strongly agree	Strongly agree
Disagree	I do not know	Strongly agree	Strongly agree
Disagree	Agree	Agree	Disagree
Agree	Disagree	Agree	Disagree
Disagree	Agree	Agree	Agree
Agree	Agree	I do not know	Disagree
Strongly agree	Agree	Agree	Strongly agree
Agree	Agree	Agree	Agree
Agree	Agree	Agree	Agree
Agree	Strongly agree	Agree	Agree
Agree	Strongly agree	Strongly agree	Agree
Disagree	I do not know	Disagree	Disagree
Agree	Agree	Agree	Agree
Agree	Agree	Agree	Agree
Disagree	Agree	Agree	Disagree
Disagree	Strongly agree	Strongly agree	Strongly agree
Agree	Agree	Agree	Agree
Strongly disagree	Strongly disagree	Disagree	Strongly disagree
I do not know	Agree	Agree	Disagree

Agree	Agree	Strongly agree	Agree
Agree	Agree	Agree	Agree
I do not know	Strongly agree	Strongly agree	Strongly agree
Strongly agree	Strongly agree	Strongly agree	Strongly agree
Strongly agree	Agree	Strongly agree	Strongly agree
Agree	Agree	Strongly agree	Agree
Agree	Agree	Disagree	Strongly agree
Strongly agree	Strongly agree	Strongly agree	Strongly agree
Strongly agree	Agree	Strongly agree	Agree
Disagree	Disagree	Agree	Strongly agree
I do not know	Agree	Strongly agree	Strongly agree
Agree	Agree	Agree	I do not know
Strongly agree	Agree	Disagree	Strongly agree
Strongly agree	Strongly agree	Strongly agree	Agree
Agree	Agree	Agree	Agree
Agree	Agree	I do not know	Agree
Strongly agree	Strongly agree	Strongly agree	Strongly agree
Agree	Disagree	Disagree	Disagree
Disagree	Disagree	Agree	Disagree
I do not know	Agree	Agree	Agree
I do not know	Disagree	Agree	Agree
Strongly agree	Strongly agree	Strongly agree	Strongly agree
I do not know	Strongly agree	Disagree	Strongly agree
Agree	Agree	I do not know	Agree
Disagree	Agree	Strongly agree	Strongly agree
Agree	Agree	Disagree	Disagree
Disagree	Disagree	Agree	Strongly disagree
Strongly disagree	Strongly disagree	Strongly disagree	Strongly disagree
I do not know	Strongly agree	I do not know	Agree
Strongly disagree	Strongly disagree	Agree	Agree
Agree	Strongly agree	Strongly agree	Strongly agree
Agree	Strongly agree	Strongly agree	Agree
Agree	Agree	Agree	Agree
Agree	Strongly agree	Disagree	Disagree
Agree	Agree	Strongly agree	Strongly agree
Agree	Disagree	Agree	Agree
Disagree	Agree	Agree	Agree
Strongly agree	I do not know	Agree	Agree
Agree	Agree	Agree	Strongly agree
Agree	Agree	Agree	Agree
Agree	Strongly agree	Strongly agree	Agree
Strongly disagree	Strongly disagree	Agree	Disagree
Agree	Strongly agree	Agree	Agree
Agree	Agree	Agree	Agree
Disagree	Disagree	Disagree	Disagree
Disagree	Agree	Strongly agree	Agree
Agree	Agree	Strongly agree	Agree
Agree	Agree	Agree	Agree
Strongly agree	Strongly agree	Agree	Agree
Strongly disagree	Disagree	Agree	I do not know
I do not know	Agree	Disagree	Agree
Agree	Strongly agree	Strongly agree	Strongly agree
I do not know	I do not know	Strongly disagree	Agree
Agree	Strongly agree	Strongly agree	Strongly agree
Agree	Strongly agree	Agree	Disagree

Agree	Agree	I do not know	Agree
Strongly agree	Strongly agree	Strongly agree	Strongly agree
Disagree	Disagree	Agree	I do not know
Agree	Strongly agree	Strongly agree	Agree
I do not know	I do not know	Agree	Agree
I do not know	Agree	Agree	I do not know
Disagree	I do not know	Agree	I do not know
Strongly agree	Strongly agree	Agree	Agree
Strongly agree	Strongly agree	Disagree	Agree
Disagree	Agree	Disagree	Disagree
Disagree	Agree	Strongly agree	I do not know
Agree	Agree	Agree	Agree
Strongly agree	Strongly agree	Strongly agree	Agree
Disagree	Agree	Disagree	Disagree
Agree	Agree	Agree	Agree
Disagree	Agree	Agree	Agree
Disagree	Agree	Agree	Agree
Agree	Agree	Agree	I do not know
I do not know	Agree	Disagree	Agree
Agree	Agree	I do not know	Agree
Agree	Agree	Agree	Agree
Agree	Agree	Agree	Agree
Agree	Strongly agree	Disagree	I do not know
Agree	Agree	Strongly agree	Strongly agree
Agree	Strongly agree	Strongly agree	Agree
I do not know	Disagree	Strongly disagree	Agree
Agree	Agree	Disagree	Agree
Strongly agree	Strongly agree	Strongly agree	Strongly agree
Agree	Agree	I do not know	Strongly agree
I do not know	I do not know	Agree	Disagree
Strongly agree	Agree	Strongly agree	Agree
Agree	Agree	Agree	Agree
I do not know	Agree	Strongly agree	Agree
I do not know	Agree	Strongly agree	Strongly agree
Agree	Agree	Strongly agree	Agree
Agree	Strongly disagree	Strongly disagree	Strongly disagree
Agree	Disagree	I do not know	Strongly disagree
I do not know	Agree	Strongly agree	Agree
Agree	Agree	Strongly agree	Agree
I do not know	Agree	Strongly agree	Agree
Agree	Agree	Agree	Agree
I do not know	I do not know	Strongly agree	Strongly agree
Agree	Agree	Agree	Agree
Disagree	Disagree	Disagree	Strongly disagree
Disagree	Strongly disagree	Strongly disagree	Strongly disagree
Agree	Agree	Strongly agree	Agree
Disagree	Disagree	Agree	Disagree
Agree	Strongly agree	Agree	Strongly agree
Agree	Strongly agree	Agree	Agree
Disagree	Agree	Agree	I do not know
Strongly agree	Strongly agree	Agree	Agree
Agree	Strongly agree	Agree	Agree
I do not know	Strongly agree	Strongly disagree	Strongly agree
Disagree	I do not know	Agree	Strongly agree
Disagree	Agree	Strongly disagree	Agree

Strongly agree	Strongly agree	Strongly agree	Strongly agree
Agree	I do not know	Agree	Agree
Agree	Agree	Agree	Agree
Strongly agree	Strongly agree	Strongly agree	Disagree
Disagree	I do not know	Strongly agree	Strongly agree
Agree	Agree	Disagree	Disagree
Disagree	Disagree	Disagree	Disagree
Disagree	Agree	I do not know	Agree
Agree	Agree	Strongly agree	Agree
Agree	Agree	Agree	Agree
Disagree	Strongly disagree	I do not know	Agree
Agree	Agree	Strongly agree	Agree
Disagree	Agree	Agree	Agree
Strongly agree	Agree	Agree	Disagree
Disagree	Strongly agree	Strongly agree	Agree
Agree	Strongly agree	Strongly agree	Strongly agree
Agree	Strongly agree	Strongly agree	Agree
Disagree	Agree	Agree	Strongly agree
Disagree	Agree	Strongly agree	Agree
Disagree	Disagree	Agree	Disagree
Agree	Agree	I do not know	Strongly agree
Strongly agree	Strongly agree	Strongly agree	Strongly agree
Disagree	Disagree	Disagree	Disagree
Agree	Strongly agree	Strongly agree	Strongly agree
Agree	Agree	Disagree	I do not know
Agree	Agree	Agree	Agree
Strongly agree	Strongly agree	Strongly agree	Strongly agree
Disagree	Agree	Agree	Agree
Agree	Disagree	Strongly agree	Strongly agree
Disagree	Agree	Disagree	Strongly agree
Disagree	Strongly agree	Agree	Agree
Agree	Disagree	Agree	Agree
Agree	Agree	I do not know	Agree
I do not know	Agree	I do not know	Agree
I do not know	Disagree	Strongly disagree	Disagree
Disagree	Agree	Agree	I do not know
I do not know	Strongly disagree	Agree	Agree
Agree	Disagree	I do not know	I do not know
Agree	Agree	Agree	Agree
Agree	Agree	Strongly agree	Agree
Disagree	Disagree	Strongly disagree	Disagree
Disagree	Agree	Strongly agree	Strongly agree
Agree	Strongly agree	Strongly agree	Strongly agree
Strongly disagree	Agree	Strongly agree	Agree
Agree	Agree	Agree	Agree
Strongly agree	Strongly agree	I do not know	Strongly agree
Agree	Strongly agree	Agree	Strongly agree
Strongly agree	I do not know	Agree	Agree
Agree	Agree	Agree	Agree
Disagree	Agree	Agree	Agree
Disagree	Strongly agree	Agree	I do not know
I do not know	Strongly agree	Strongly agree	Strongly agree
Agree	Strongly agree	Agree	Strongly agree
Disagree	Agree	Agree	Agree
I do not know	Agree	Strongly disagree	Agree

Agree	Agree	Agree	Agree
Agree	Strongly agree	Strongly agree	Agree
Agree	Agree	Agree	Agree
Agree	I do not know	Strongly agree	Strongly agree
Strongly agree	Strongly agree	Strongly agree	Agree
Agree	Agree	Strongly agree	Strongly agree
Agree	Agree	Disagree	Disagree
Strongly disagree	Agree	Agree	Disagree
Agree	Agree	Agree	Agree
Agree	Strongly agree	Strongly agree	Strongly agree
Agree	Agree	Agree	Agree
Strongly disagree	Agree	Agree	Disagree
Strongly disagree	Strongly disagree	Strongly disagree	Strongly disagree
Agree	Agree	Strongly agree	Agree
Disagree	Agree	Agree	Agree
Agree	Agree	Agree	Disagree
Agree	Agree	Disagree	Disagree
Disagree	Agree	Agree	Agree
I do not know	I do not know	Strongly disagree	I do not know
Disagree	Agree	Strongly agree	Agree
Disagree	Agree	Strongly disagree	Agree
I do not know	Agree	Disagree	Agree
Strongly agree	Strongly agree	Disagree	Strongly agree
Disagree	Agree	Agree	Disagree
Strongly agree	Agree	Agree	Agree
Agree	Agree	Agree	Agree
Agree	Strongly agree	Strongly agree	Agree
Disagree	Agree	Agree	Agree
Disagree	I do not know	Strongly agree	Agree
Agree	Strongly agree	Strongly agree	Strongly agree
I do not know	Strongly agree	Agree	Agree
Agree	Agree	Agree	Agree
Strongly disagree	I do not know	Agree	I do not know
I do not know	Agree	Strongly agree	I do not know
I do not know	Strongly agree	Strongly agree	Agree
Strongly agree	Strongly agree	Strongly agree	Strongly agree
Strongly agree	Agree	Strongly agree	Strongly agree
I do not know	Agree	Strongly disagree	Strongly disagree
Agree	Agree	I do not know	Strongly agree
Agree	Strongly agree	Strongly agree	Strongly agree
Strongly agree	Strongly agree	Strongly agree	Strongly agree
Strongly disagree	Agree	Strongly agree	Strongly agree
Agree	Disagree	Disagree	Disagree
Agree	Disagree	Disagree	I do not know
Agree	I do not know	Agree	Agree
Strongly disagree	Agree	Agree	Agree
I do not know	Agree	Strongly agree	Strongly agree
Agree	Strongly agree	Strongly agree	Strongly agree
Agree	Agree	Agree	Strongly agree
I do not know	Strongly agree	Strongly agree	I do not know
I do not know	Strongly agree	Agree	I do not know
I do not know	Strongly agree	Strongly agree	Agree
Strongly agree	Strongly agree	Strongly agree	Agree
Agree	Agree	Agree	Agree
Agree	Disagree	Disagree	Disagree

Agree	I do not know	Agree	Agree
Agree	Agree	Agree	Agree
Disagree	Disagree	Disagree	Disagree
Agree	Agree	Strongly disagree	Disagree
Agree	Agree	Strongly agree	Disagree
Disagree	Agree	Agree	Agree
Agree	Agree	Agree	Agree
Agree	Strongly agree	Agree	Strongly agree
I do not know	Agree	Agree	Agree
Agree	Agree	Agree	Agree
I do not know	Agree	Strongly agree	Agree
Agree	I do not know	Agree	Strongly agree
Agree	Strongly agree	Strongly agree	Agree
Strongly agree	Strongly agree	Strongly agree	Strongly agree
Strongly agree	Strongly agree	Agree	Agree
Agree	Agree	Strongly agree	Agree
Agree	Agree	Strongly agree	Agree
Disagree	Disagree	Disagree	Disagree
Disagree	Agree	Agree	Strongly disagree
I do not know	Agree	Agree	Agree
I do not know	I do not know	Strongly agree	Strongly agree
Agree	Strongly agree	Strongly agree	Strongly agree
Disagree	Strongly agree	Strongly agree	Disagree
Disagree	Agree	Disagree	Agree
Disagree	Disagree	Agree	Disagree
Agree	Agree	Agree	Agree
Agree	Agree	Agree	Agree
Agree	Agree	Agree	Agree
Strongly agree	Strongly agree	Strongly agree	Agree
Agree	Agree	Disagree	Agree
Disagree	Strongly agree	Agree	Agree
Agree	Agree	Strongly agree	Agree
Agree	Agree	Strongly agree	Strongly agree
Agree	Strongly agree	Strongly agree	Agree
Agree	Strongly agree	Disagree	Agree
I do not know	Agree	Disagree	Disagree
I do not know	Strongly agree	Agree	Strongly agree
Strongly agree	Strongly agree	Strongly agree	Strongly agree
Agree	Agree	Agree	Agree
Strongly disagree	Strongly disagree	Strongly disagree	Strongly disagree
Agree	I do not know	Agree	Strongly agree
I do not know	I do not know	Strongly disagree	I do not know
I do not know	I do not know	Disagree	Disagree
I do not know	Disagree	Disagree	Agree
Agree	Agree	Agree	Agree
Agree	Agree	Strongly agree	Strongly agree
Agree	Strongly agree	I do not know	Agree
Agree	Agree	Agree	Agree
Agree	Strongly agree	Agree	Agree
Agree	Agree	Strongly agree	Agree
I do not know	Agree	Strongly agree	Agree
Agree	Strongly disagree	I do not know	Agree
I do not know	I do not know	Strongly agree	Agree
Agree	Strongly agree	Strongly agree	Strongly agree
Disagree	Agree	I do not know	Agree

Strongly disagree	I do not know	Strongly disagree	I do not know
Strongly agree	Strongly agree	Strongly agree	Strongly agree
Agree	Strongly agree	Agree	I do not know
Agree	Disagree	Disagree	Disagree
Disagree	Disagree	Agree	Disagree
Disagree	Agree	Agree	Agree
Disagree	I do not know	Agree	Disagree
Disagree	Strongly agree	Disagree	Disagree
Strongly agree	Agree	Strongly agree	Agree
Strongly agree	I do not know	Strongly disagree	Strongly agree
Disagree	Disagree	Agree	Disagree
Agree	Agree	Strongly agree	Strongly agree
Strongly disagree	Strongly agree	Agree	Agree
Strongly disagree	I do not know	Strongly agree	Disagree
I do not know	Agree	Strongly agree	Agree
I do not know	Strongly disagree	Agree	I do not know
Agree	Agree	Strongly agree	Strongly agree
Agree	Agree	Agree	Agree
Agree	I do not know	Strongly agree	Agree
Agree	Agree	Agree	Agree
Strongly agree	Strongly agree	Strongly agree	Strongly agree
I do not know	Strongly disagree	Strongly disagree	I do not know
Agree	Disagree	Strongly agree	Strongly agree
Agree	I do not know	I do not know	Agree
Agree	Disagree	Disagree	Strongly agree
Disagree	Disagree	Strongly disagree	Disagree
Agree	Agree	I do not know	I do not know
Agree	Agree	Strongly agree	Agree
Strongly agree	Agree	Strongly agree	Agree
Strongly agree	Strongly disagree	Agree	Strongly agree
Strongly agree	Agree	Strongly disagree	Disagree
Disagree	Agree	Agree	Disagree
Disagree	Disagree	Disagree	Disagree
Agree	Agree	I do not know	I do not know
I do not know	Agree	Agree	Agree
I do not know	Agree	Disagree	I do not know
I do not know	Agree	Agree	Agree
Disagree	Agree	Agree	Disagree
Agree	Agree	Agree	Agree
Disagree	Agree	Agree	Agree
Strongly agree	Agree	Disagree	Disagree
I do not know	Agree	Agree	Agree
Agree	Agree	Agree	Agree
Strongly agree	Strongly agree	Strongly agree	Strongly agree
Agree	Agree	Agree	Agree
Agree	Agree	Agree	Agree
Disagree	Agree	Strongly agree	Agree
Agree	Agree	Disagree	Disagree
I do not know	Agree	Strongly agree	Agree
I do not know	Agree	Disagree	Agree
I do not know	Agree	I do not know	Agree
Agree	Strongly disagree	I do not know	I do not know
Agree	Strongly agree	Strongly agree	Agree
Strongly agree	Strongly agree	Strongly agree	Strongly agree
Disagree	Agree	Agree	Disagree

Agree	Strongly agree	Strongly agree	Strongly agree
Disagree	Disagree	Disagree	Disagree
Strongly disagree	Disagree	Strongly agree	Strongly agree
Disagree	Disagree	Strongly agree	I do not know
I do not know	Agree	Strongly disagree	I do not know
Strongly agree	Strongly agree	Strongly agree	Strongly agree
Disagree	Agree	Strongly disagree	Disagree
Agree	Agree	Agree	Agree
Strongly agree	Strongly agree	Agree	Agree
Strongly disagree	I do not know	Strongly agree	Strongly agree
Agree	Strongly agree	Strongly agree	Agree
Agree	Agree	Agree	Agree
I do not know	Disagree	Agree	I do not know
Agree	Strongly agree	Strongly agree	Agree
I do not know	Agree	Agree	Disagree
I do not know	Agree	I do not know	Agree
I do not know	Agree	Agree	Agree

One of the reasons for viol	One of the reasons for viol	One of the reasons for viol	One of the reasons for viol
Disagree	Disagree	Disagree	I do not know
I do not know	Disagree	Strongly disagree	Strongly disagree
Strongly agree	Agree	Agree	Strongly agree
Strongly agree	Agree	Agree	Agree
Disagree	Disagree	Disagree	Disagree
I do not know	Disagree	Agree	Agree
Agree	Agree	Strongly disagree	Disagree
Disagree	Disagree	I do not know	Agree
Agree	Agree	Agree	Agree
Strongly agree	I do not know	Agree	Agree
Disagree	Agree	Disagree	Agree
Agree	Disagree	Disagree	Disagree
Disagree	Strongly agree	Agree	Strongly agree
Strongly agree	Strongly agree	Agree	Strongly agree
Strongly agree	Strongly agree	Strongly agree	Strongly agree
Agree	Agree	Disagree	Agree
Agree	Agree	Agree	Agree
Disagree	Disagree	Agree	Agree
Strongly agree	Strongly agree	Strongly agree	Strongly agree
Agree	Strongly disagree	I do not know	Strongly agree
Agree	Strongly agree	Strongly agree	Strongly agree
Agree	Agree	Agree	Agree
Agree	Agree	I do not know	Strongly agree
Strongly agree	Strongly agree	Agree	I do not know
Strongly agree	Strongly agree	Agree	I do not know
Disagree	I do not know	Agree	Disagree
Agree	I do not know	I do not know	Strongly disagree
Disagree	Disagree	Disagree	Disagree
Disagree	I do not know	Strongly disagree	Strongly disagree
Strongly disagree	Disagree	I do not know	Disagree
Agree	Agree	Disagree	Agree
I do not know	I do not know	I do not know	Agree
Strongly agree	Disagree	Disagree	Agree
Agree	Agree	Agree	Agree
Strongly agree	Strongly agree	Disagree	Strongly agree
Strongly disagree	Strongly disagree	Strongly disagree	Strongly disagree
Agree	Agree	Disagree	Disagree
Disagree	Disagree	Disagree	Disagree
Strongly agree	Strongly agree	Agree	Strongly agree
Agree	Strongly agree	Disagree	Agree
Agree	Strongly agree	Agree	Agree
Strongly agree	Strongly agree	Strongly agree	I do not know
Strongly agree	Strongly agree	Strongly agree	Strongly agree
Strongly agree	Disagree	Disagree	Agree
Strongly agree	Strongly agree	Disagree	Agree
Strongly agree	Strongly disagree	Strongly disagree	Strongly agree
Agree	Agree	Agree	Agree
Agree	Agree	Disagree	Agree

Strongly agree	Disagree	Disagree	Agree
Agree	Agree	Disagree	I do not know
I do not know	I do not know	I do not know	I do not know
Strongly agree	Strongly agree	Agree	Strongly agree
I do not know	Disagree	Disagree	Agree
Disagree	Agree	Disagree	Agree
I do not know	Disagree	Disagree	Disagree
I do not know	Agree	I do not know	I do not know
Agree	Agree	Disagree	Disagree
Agree	I do not know	I do not know	I do not know
Agree	Agree	Disagree	Disagree
Strongly agree	Strongly agree	Strongly agree	Strongly agree
Agree	Agree	Agree	I do not know
Strongly disagree	I do not know	Strongly disagree	Strongly disagree
Strongly agree	Strongly agree	I do not know	Agree
Agree	Agree	Agree	Agree
Agree	Agree	Agree	Agree
Strongly agree	Strongly agree	Agree	Agree
Agree	Agree	Agree	Agree
Agree	Agree	Agree	Agree
Agree	Disagree	Agree	Agree
Agree	Agree	I do not know	Strongly agree
Agree	Agree	Disagree	Agree
Strongly agree	Agree	Agree	Agree
Strongly agree	I do not know	I do not know	Agree
Strongly agree	Strongly agree	I do not know	Strongly agree
Agree	Disagree	Strongly disagree	Strongly disagree
Agree	Strongly agree	Agree	Agree
Agree	Agree	Agree	Agree
Agree	Agree	Disagree	Disagree
Agree	Agree	Agree	Agree
Disagree	Disagree	Disagree	Disagree
Agree	Agree	Agree	Agree
Agree	Disagree	Disagree	Agree
Agree	Agree	Agree	Agree
Agree	Agree	Disagree	Agree
Disagree	Agree	Disagree	Agree
Disagree	Strongly disagree	I do not know	Disagree
Agree	Agree	I do not know	Agree
I do not know	Disagree	Disagree	Agree
Strongly agree	Strongly agree	Strongly agree	Strongly agree
Strongly disagree	Disagree	Disagree	Disagree
Agree	Agree	Agree	I do not know
I do not know	Agree	Agree	I do not know
Agree	Agree	Agree	Agree
Agree	Agree	Agree	Agree
Strongly agree	Disagree	Disagree	Agree
Strongly agree	Strongly agree	Strongly agree	Strongly agree
I do not know	I do not know	Agree	I do not know
Strongly agree	Agree	Agree	Strongly agree
Agree	Strongly agree	Disagree	Agree
Strongly agree	Strongly agree	Agree	Agree
Disagree	I do not know	I do not know	I do not know
Strongly agree	Strongly agree	Strongly agree	Strongly agree
I do not know	Disagree	Disagree	Disagree

Disagree	Disagree	I do not know	Disagree
Disagree	I do not know	Disagree	I do not know
Disagree	Agree	Disagree	I do not know
Agree	Agree	I do not know	Agree
Disagree	I do not know	I do not know	I do not know
Strongly agree	Agree	I do not know	Agree
Agree	Agree	Agree	Agree
Disagree	Disagree	Disagree	Agree
Agree	Agree	Agree	Agree
Strongly agree	Strongly agree	Agree	Agree
I do not know	Agree	I do not know	Agree
Strongly agree	Disagree	I do not know	Agree
Agree	Agree	Agree	Agree
Disagree	Disagree	Disagree	I do not know
Agree	I do not know	I do not know	I do not know
Agree	Agree	Disagree	Agree
Agree	Strongly agree	I do not know	I do not know
Agree	Disagree	Disagree	Agree
Agree	Agree	Agree	Agree
Agree	Agree	Disagree	Agree
Agree	I do not know	I do not know	Agree
Agree	I do not know	I do not know	Disagree
I do not know	Disagree	Disagree	Agree
Agree	Disagree	Agree	Agree
Strongly agree	Agree	I do not know	Strongly agree
Agree	Disagree	Agree	Agree
Agree	Agree	Disagree	Disagree
Strongly disagree	Strongly agree	Agree	Agree
Agree	I do not know	Disagree	I do not know
Agree	I do not know	Agree	Agree
Strongly disagree	I do not know	Agree	Disagree
Strongly agree	Strongly agree	Strongly agree	Strongly agree
Agree	Agree	Agree	Agree
Strongly disagree	Strongly disagree	Strongly disagree	Strongly disagree
Strongly agree	Disagree	Disagree	Agree
Agree	Agree	Disagree	Agree
Agree	Agree	Disagree	Agree
Agree	Agree	Disagree	Agree
Strongly agree	Strongly agree	I do not know	Agree
Disagree	Disagree	Disagree	Disagree
Strongly agree	Agree	Disagree	Agree
Disagree	Disagree	Disagree	Agree
Agree	Agree	Agree	Agree
Agree	Agree	Agree	Agree
Agree	Agree	Disagree	Agree
Agree	Agree	Agree	Disagree
Strongly agree	Strongly agree	Agree	Agree
Agree	Disagree	Disagree	Disagree
Disagree	Strongly disagree	Strongly disagree	Agree
Agree	Agree	Disagree	Agree
Agree	Disagree	Disagree	Disagree
Strongly agree	Strongly agree	Disagree	Strongly agree
Agree	Agree	Agree	Agree
Disagree	Strongly disagree	Disagree	Strongly disagree
Agree	Agree	I do not know	Agree

Agree	Disagree	Disagree	Agree
Agree	Agree	I do not know	Agree
Agree	Agree	I do not know	Agree
Strongly agree	Strongly agree	Agree	Strongly agree
Agree	Strongly agree	Disagree	Strongly agree
Agree	I do not know	I do not know	Strongly agree
Agree	Agree	I do not know	Strongly agree
Strongly agree	Strongly agree	Strongly agree	Strongly agree
Strongly agree	Agree	Strongly agree	I do not know
Strongly agree	I do not know	Agree	Agree
Agree	Agree	Strongly disagree	Agree
I do not know	I do not know	I do not know	I do not know
Agree	Agree	Disagree	Agree
Strongly agree	Strongly agree	Agree	Strongly agree
Agree	Agree	Agree	Agree
I do not know	I do not know	Strongly disagree	Strongly agree
Strongly agree	Strongly agree	Strongly agree	Strongly agree
I do not know	Agree	Disagree	Agree
Agree	Agree	Disagree	Agree
Agree	Agree	I do not know	Agree
Agree	Agree	I do not know	I do not know
Strongly agree	Strongly agree	Strongly agree	Strongly agree
Strongly agree	Disagree	I do not know	Strongly agree
Agree	Agree	Agree	Agree
Agree	Agree	Disagree	Disagree
Strongly disagree	Strongly disagree	I do not know	Agree
Strongly disagree	Agree	Disagree	Strongly disagree
Strongly disagree	Strongly disagree	Strongly disagree	Strongly disagree
Strongly agree	Strongly agree	I do not know	Disagree
Agree	Agree	Agree	Strongly disagree
Strongly agree	Strongly agree	Agree	Agree
Strongly agree	Agree	I do not know	Agree
Strongly agree	Disagree	Disagree	Disagree
Agree	Agree	Strongly agree	Agree
Strongly agree	Agree	Agree	Agree
I do not know	Agree	Disagree	I do not know
Agree	Agree	Disagree	Agree
I do not know	Disagree	Disagree	Agree
Strongly agree	Strongly agree	Strongly agree	Strongly agree
Agree	Agree	Disagree	Agree
Agree	Agree	Agree	Agree
Strongly agree	Disagree	Disagree	Agree
Agree	Agree	Agree	Agree
Agree	Agree	Agree	Agree
Agree	Disagree	Disagree	Disagree
Strongly agree	Agree	Disagree	Strongly agree
Agree	Agree	Agree	Agree
I do not know	Agree	Disagree	Disagree
Strongly agree	Strongly agree	I do not know	I do not know
Agree	Disagree	Strongly disagree	Agree
Agree	Agree	I do not know	I do not know
Strongly agree	Agree	Disagree	Strongly agree
Strongly disagree	Strongly disagree	Strongly disagree	Strongly disagree
Agree	Agree	Disagree	Agree
Agree	Strongly disagree	Disagree	Agree

Agree	Agree	I do not know	Agree
Strongly agree	Strongly agree	Agree	Strongly agree
Agree	Disagree	Agree	Agree
Strongly agree	Strongly agree	I do not know	Agree
Strongly agree	Agree	I do not know	Strongly agree
I do not know	I do not know	I do not know	I do not know
Agree	I do not know	Disagree	Agree
Strongly agree	Agree	I do not know	Agree
Strongly agree	Disagree	Agree	Disagree
Agree	Agree	Disagree	Disagree
Strongly agree	Agree	I do not know	Agree
Disagree	Agree	Disagree	Agree
Strongly agree	I do not know	I do not know	Strongly agree
Disagree	I do not know	Disagree	Agree
Agree	Disagree	Disagree	Disagree
Agree	Disagree	Disagree	Agree
Agree	Agree	Disagree	I do not know
Strongly agree	Agree	Agree	Agree
I do not know	Agree	I do not know	Agree
Agree	Agree	I do not know	Agree
Agree	Disagree	I do not know	Agree
Agree	Agree	Agree	Agree
I do not know	Disagree	Agree	I do not know
Strongly agree	Strongly agree	I do not know	Strongly agree
Agree	Agree	Disagree	Strongly agree
Strongly disagree	Strongly disagree	Strongly disagree	Disagree
Strongly disagree	Agree	Disagree	Agree
Strongly agree	Strongly agree	Strongly agree	Strongly agree
Strongly agree	Agree	I do not know	Agree
Agree	Disagree	Agree	Agree
Agree	I do not know	Agree	Agree
Agree	Agree	Disagree	Agree
Agree	Agree	Agree	Strongly agree
Strongly agree	Agree	Agree	Agree
Agree	Agree	I do not know	Agree
Agree	Strongly disagree	I do not know	Strongly disagree
I do not know	Agree	I do not know	Agree
Agree	Agree	I do not know	Agree
Agree	Agree	Agree	Agree
Agree	Agree	Disagree	Agree
Agree	Agree	Agree	Strongly agree
Agree	Agree	I do not know	Agree
Agree	Agree	Agree	Agree
Agree	Strongly disagree	Strongly disagree	Agree
Disagree	Strongly disagree	Strongly disagree	Disagree
Strongly agree	Agree	Agree	I do not know
Agree	Agree	I do not know	Agree
Strongly agree	Strongly agree	Agree	Agree
Strongly agree	I do not know	Disagree	Disagree
I do not know	Agree	Disagree	Disagree
Agree	Disagree	Disagree	Disagree
Strongly agree	Agree	Disagree	Agree
I do not know	Strongly agree	Agree	I do not know
Agree	Strongly agree	I do not know	Agree
Agree	Disagree	Disagree	Disagree

Strongly agree	Strongly agree	Strongly agree	Strongly agree
Agree	Agree	Disagree	I do not know
Agree	I do not know	I do not know	I do not know
I do not know	Disagree	Agree	Agree
Agree	Agree	Disagree	Agree
Agree	Disagree	Disagree	Disagree
Disagree	Disagree	Disagree	Disagree
Agree	Agree	Agree	Agree
Strongly agree	Agree	Agree	Strongly agree
Agree	Agree	Agree	Agree
Strongly agree	Agree	Strongly agree	Strongly disagree
Strongly agree	Agree	Agree	Agree
Agree	Agree	I do not know	Agree
Agree	Agree	Agree	Strongly agree
Agree	Agree	Strongly disagree	Agree
Strongly agree	Strongly agree	Strongly agree	Strongly agree
Strongly agree	Strongly agree	Agree	Strongly agree
Agree	Strongly disagree	Agree	Agree
Agree	Agree	Agree	Agree
Agree	Disagree	Disagree	Disagree
Agree	Strongly disagree	Agree	Agree
Strongly agree	Strongly agree	Strongly agree	Strongly agree
Disagree	Disagree	Disagree	Disagree
Strongly agree	Strongly agree	Strongly agree	Agree
Agree	I do not know	I do not know	Agree
Agree	Agree	Agree	Agree
Strongly agree	Strongly agree	Strongly agree	Strongly agree
Agree	Disagree	Disagree	Agree
Strongly agree	Agree	Agree	Agree
Agree	I do not know	Disagree	Agree
Strongly agree	Agree	Agree	Agree
Agree	Agree	I do not know	Strongly agree
I do not know	Agree	I do not know	Agree
Agree	Agree	I do not know	Agree
I do not know	Disagree	I do not know	I do not know
Agree	Disagree	Agree	Disagree
Agree	Agree	I do not know	Agree
Agree	Agree	I do not know	Agree
Disagree	Disagree	Disagree	Agree
Strongly agree	Agree	Agree	Agree
Agree	Disagree	Disagree	Disagree
Strongly agree	Agree	I do not know	Agree
Strongly agree	Agree	I do not know	Strongly agree
Strongly agree	Strongly agree	I do not know	Agree
Agree	Agree	Agree	Agree
Strongly agree	Agree	I do not know	Strongly disagree
Agree	Agree	Strongly agree	Strongly agree
I do not know	Agree	I do not know	Agree
Agree	Agree	Strongly agree	Agree
Agree	Agree	Agree	Agree
Agree	Disagree	Agree	Agree
Disagree	Strongly agree	Disagree	Agree
Strongly agree	Agree	Disagree	Disagree
Disagree	Agree	Agree	Agree
Agree	I do not know	Agree	Agree

Agree	Agree	Agree	Agree
Strongly agree	Strongly agree	Strongly agree	Strongly agree
Agree	Agree	I do not know	Agree
Agree	Strongly agree	Disagree	Strongly agree
Strongly agree	Agree	I do not know	Strongly agree
Strongly agree	Strongly agree	Strongly agree	Strongly agree
Strongly agree	Agree	Strongly disagree	Disagree
Agree	Agree	Strongly disagree	I do not know
Agree	Agree	I do not know	Agree
Strongly agree	Strongly agree	Strongly agree	Strongly agree
Agree	Agree	Agree	Agree
Agree	Disagree	Agree	Agree
Strongly disagree	Strongly disagree	Strongly disagree	Strongly disagree
Agree	Agree	Strongly agree	Agree
Agree	Disagree	Disagree	Agree
Strongly agree	Strongly agree	Disagree	Agree
Agree	Disagree	Disagree	I do not know
Agree	Agree	Disagree	Agree
Agree	Disagree	I do not know	I do not know
Agree	Strongly agree	Agree	Agree
Strongly agree	Strongly disagree	I do not know	Agree
Agree	Disagree	Disagree	Agree
I do not know	Strongly agree	Strongly agree	Disagree
Agree	Agree	Disagree	Disagree
I do not know	Disagree	I do not know	I do not know
Agree	Agree	Agree	Agree
Strongly agree	Agree	I do not know	Agree
Agree	I do not know	Disagree	Agree
Agree	Disagree	I do not know	Agree
Strongly agree	Strongly agree	Agree	Strongly agree
Agree	Agree	Strongly agree	Strongly agree
Agree	Agree	Agree	Agree
Agree	Strongly agree	Agree	Agree
Disagree	Disagree	Disagree	Agree
Strongly agree	Strongly agree	Agree	Strongly agree
Agree	Strongly agree	I do not know	Agree
I do not know	Agree	I do not know	Agree
I do not know	Disagree	Disagree	Agree
Strongly agree	Agree	I do not know	Agree
Strongly agree	Strongly agree	Agree	Strongly agree
Strongly agree	Strongly agree	Agree	Strongly agree
Agree	Agree	Strongly disagree	Agree
Disagree	I do not know	I do not know	Disagree
I do not know	Disagree	Disagree	Agree
Agree	Agree	Agree	Agree
Agree	Agree	Disagree	Agree
Strongly agree	Strongly agree	I do not know	Strongly agree
Strongly agree	Strongly agree	Agree	Strongly agree
Strongly agree	Strongly agree	Agree	Agree
Strongly agree	I do not know	Disagree	Strongly agree
Agree	I do not know	I do not know	Agree
Strongly agree	Agree	I do not know	Strongly agree
Agree	Agree	Agree	Agree
Agree	Agree	Agree	Agree
Disagree	Agree	Agree	Agree

Strongly agree	I do not know	Agree	Strongly agree
Agree	Agree	I do not know	Agree
I do not know	I do not know	I do not know	I do not know
Agree	Agree	Agree	Agree
Disagree	Agree	Agree	Agree
I do not know	Agree	Agree	Agree
Agree	Agree	Agree	Agree
Strongly agree	Agree	Agree	Agree
Agree	I do not know	I do not know	Agree
I do not know	Agree	Agree	Agree
Agree	Agree	Disagree	Agree
I do not know	Agree	Strongly agree	Strongly agree
Agree	Agree	Strongly disagree	Agree
Disagree	I do not know	Disagree	Disagree
Agree	Strongly disagree	Strongly disagree	I do not know
Strongly agree	Disagree	Agree	Agree
Strongly agree	Agree	Agree	Agree
I do not know	Disagree	I do not know	Disagree
Strongly disagree	I do not know	Disagree	Agree
Agree	Strongly disagree	I do not know	Agree
Strongly agree	Agree	I do not know	Agree
Agree	Agree	Agree	Strongly agree
Agree	Agree	Disagree	Agree
Agree	I do not know	Disagree	Agree
I do not know	I do not know	I do not know	Disagree
Agree	I do not know	I do not know	Disagree
Agree	Agree	Agree	Agree
Agree	Disagree	Agree	I do not know
Strongly agree	Agree	Agree	Agree
Strongly agree	Disagree	Agree	Disagree
Agree	Agree	Disagree	Agree
Agree	Agree	Agree	Agree
Strongly agree	Strongly agree	Agree	Agree
Agree	Agree	I do not know	Agree
Disagree	Agree	Disagree	Agree
Agree	Agree	Disagree	Disagree
Agree	Agree	Agree	Agree
Strongly agree	Strongly agree	Strongly agree	Strongly agree
Agree	Agree	Strongly disagree	Strongly disagree
Strongly disagree	Strongly disagree	Strongly disagree	Strongly disagree
Agree	I do not know	I do not know	I do not know
Agree	I do not know	I do not know	I do not know
Disagree	Disagree	I do not know	Disagree
I do not know	Agree	Agree	Disagree
Agree	Agree	Agree	Agree
Agree	Agree	Agree	Agree
Agree	Agree	I do not know	Strongly agree
Agree	Agree	Agree	Agree
Strongly agree	Strongly agree	Disagree	Agree
Agree	Agree	Agree	Agree
Disagree	Agree	Disagree	I do not know
Strongly disagree	Disagree	Strongly agree	Strongly agree
Strongly agree	Strongly agree	Agree	Strongly agree
Strongly agree	Agree	I do not know	Agree
I do not know	I do not know	I do not know	I do not know

Strongly disagree	Disagree	Disagree	I do not know
Agree	Strongly agree	Agree	Strongly agree
Disagree	I do not know	I do not know	Agree
Disagree	Disagree	Agree	Disagree
Agree	Disagree	Disagree	Agree
Strongly agree	Agree	Disagree	Agree
Strongly disagree	Disagree	Disagree	I do not know
I do not know	I do not know	Agree	Disagree
Strongly agree	Strongly agree	Agree	Agree
Strongly agree	Strongly agree	Strongly agree	Agree
Agree	Strongly disagree	I do not know	Disagree
Strongly agree	Agree	Disagree	Agree
Agree	Agree	Strongly disagree	Disagree
Disagree	Agree	Agree	Strongly agree
Strongly agree	I do not know	I do not know	Agree
I do not know	I do not know	I do not know	Agree
Agree	Agree	I do not know	Agree
Agree	Agree	Agree	Agree
Strongly agree	Agree	Disagree	Agree
Agree	Agree	I do not know	Disagree
Strongly agree	Agree	Agree	Strongly agree
Disagree	Strongly disagree	Strongly disagree	Strongly disagree
Agree	Strongly agree	Agree	Strongly agree
Agree	Agree	Agree	Agree
Agree	Agree	Agree	Agree
Agree	Strongly disagree	Strongly disagree	Disagree
Agree	I do not know	Agree	Agree
Agree	Agree	Agree	Strongly agree
Strongly agree	Strongly agree	Agree	Strongly agree
Strongly agree	Agree	Strongly agree	Strongly disagree
Strongly agree	Disagree	Disagree	Agree
Disagree	Disagree	Agree	Agree
Disagree	Disagree	Disagree	Agree
Agree	I do not know	Agree	Disagree
Agree	Agree	Agree	Agree
Disagree	Disagree	Disagree	Disagree
Agree	Agree	I do not know	Agree
Strongly agree	Agree	Agree	Agree
Agree	Agree	Agree	Agree
Agree	Agree	Disagree	Agree
Agree	Agree	Disagree	Disagree
Agree	Strongly agree	Agree	Agree
Agree	Agree	Agree	Disagree
Strongly agree	Strongly agree	Strongly agree	Strongly agree
Agree	Agree	Agree	Agree
Agree	Agree	I do not know	Agree
Agree	I do not know	I do not know	Agree
Agree	Disagree	Disagree	Agree
Agree	Strongly agree	Agree	Agree
Disagree	Agree	Agree	Strongly agree
Strongly agree	I do not know	I do not know	Agree
Disagree	Disagree	Agree	Agree
Agree	Strongly agree	Agree	Strongly agree
Strongly agree	Strongly agree	Strongly agree	Strongly agree
Agree	Strongly disagree	Disagree	Disagree

Agree	Agree	I do not know	Agree
Strongly disagree	Disagree	Disagree	Disagree
Strongly agree	Agree	Disagree	Disagree
Strongly agree	Strongly agree	Strongly agree	Strongly agree
Agree	Disagree	Disagree	Strongly disagree
Strongly agree	Strongly agree	Strongly agree	Strongly agree
Disagree	Disagree	Disagree	Strongly disagree
Agree	Agree	Agree	Agree
Agree	Agree	I do not know	Agree
Strongly agree	Strongly agree	Strongly agree	Strongly agree
Agree	Disagree	Disagree	Agree
Agree	Agree	Agree	Agree
Disagree	Strongly disagree	Disagree	Disagree
Strongly agree	Agree	Agree	Agree
I do not know	Agree	Disagree	Agree
Agree	I do not know	I do not know	Agree
I do not know	I do not know	Agree	Agree

One of the reasons for viol	One of the reasons for viol	One of the reasons for viol	One of the reasons for viol
Agree	Agree	Agree	Strongly agree
I do not know	Agree	Strongly disagree	I do not know
Agree	Agree	Strongly agree	Agree
Agree	Agree	Disagree	Disagree
Disagree	Agree	Disagree	Disagree
Agree	Agree	Strongly disagree	Strongly agree
Disagree	Disagree	Agree	Agree
Disagree	Disagree	Disagree	Disagree
Agree	Agree	Agree	Agree
Agree	I do not know	Agree	Agree
Agree	Disagree	Disagree	Disagree
Agree	Disagree	Disagree	Disagree
Agree	Strongly agree	Agree	Agree
Strongly agree	Agree	Agree	Agree
Strongly agree	Strongly agree	Agree	Strongly agree
Agree	Agree	Agree	Agree
Agree	Agree	Agree	Agree
Disagree	Agree	I do not know	Disagree
Strongly agree	Strongly agree	Strongly agree	Strongly agree
Agree	Strongly disagree	Strongly agree	Strongly agree
Strongly agree	Strongly agree	Strongly agree	Strongly agree
Agree	Agree	I do not know	Agree
Strongly disagree	Agree	Strongly agree	Strongly agree
Agree	Disagree	I do not know	Disagree
Agree	Disagree	I do not know	Disagree
Disagree	I do not know	Agree	Disagree
Strongly disagree	Agree	Disagree	Agree
Disagree	Disagree	Disagree	Disagree
Strongly disagree	Strongly disagree	I do not know	Agree
Strongly disagree	Disagree	I do not know	Strongly agree
Agree	Disagree	Disagree	Strongly disagree
I do not know	I do not know	Disagree	Disagree
Agree	I do not know	Agree	Agree
Agree	Agree	Agree	Strongly agree
Agree	Agree	Agree	Agree
Strongly disagree	Strongly disagree	Strongly disagree	Strongly disagree
Agree	Agree	Agree	Agree
Disagree	Disagree	I do not know	Disagree
Agree	Agree	Agree	Agree
Agree	Strongly disagree	Agree	Agree
Strongly agree	Strongly agree	Agree	Strongly agree
Agree	Agree	Strongly agree	Agree
Strongly agree	Strongly agree	Strongly agree	Strongly agree
Disagree	I do not know	I do not know	Disagree
Strongly agree	Disagree	Agree	Disagree
Agree	Strongly agree	Strongly agree	Strongly agree
Agree	Strongly agree	Strongly agree	Agree
Agree	I do not know	Disagree	Agree

Disagree	Disagree	Agree	Strongly agree
I do not know	Disagree	Agree	I do not know
I do not know	I do not know	I do not know	I do not know
I do not know	Agree	Agree	Agree
Agree	Agree	I do not know	Agree
Agree	Agree	Agree	Strongly agree
I do not know	Agree	I do not know	Agree
Agree	I do not know	I do not know	Agree
Agree	I do not know	Disagree	Agree
Agree	Agree	Agree	I do not know
Agree	Strongly agree	Disagree	Agree
Strongly agree	Strongly agree	Strongly agree	Agree
Strongly agree	Agree	Agree	Disagree
I do not know	I do not know	Strongly disagree	Agree
Agree	Agree	I do not know	I do not know
Agree	Agree	Agree	Agree
Agree	Agree	I do not know	Agree
Agree	Disagree	Agree	Disagree
Strongly agree	Agree	Strongly agree	Strongly agree
Agree	Agree	I do not know	Agree
Agree	Agree	Agree	Disagree
Agree	I do not know	Agree	I do not know
Disagree	I do not know	Agree	Disagree
Agree	Strongly agree	Agree	Agree
Strongly agree	Agree	Agree	Agree
Strongly agree	Agree	Strongly agree	Agree
Disagree	I do not know	Agree	Strongly disagree
Agree	Agree	Agree	Agree
Agree	Agree	Agree	Agree
I do not know	I do not know	I do not know	I do not know
Agree	Agree	Agree	Agree
Disagree	Disagree	Disagree	Disagree
Agree	Agree	Agree	Agree
Agree	Agree	Disagree	Agree
I do not know	Agree	Agree	Agree
Agree	Agree	Agree	Strongly agree
Strongly agree	Agree	Agree	Agree
I do not know	Agree	Disagree	Agree
Agree	Agree	Agree	Agree
Agree	Agree	Disagree	Disagree
I do not know	Strongly agree	Agree	Strongly agree
Disagree	Disagree	Agree	Strongly disagree
Agree	Agree	Strongly disagree	Agree
I do not know	Agree	Disagree	Disagree
Agree	I do not know	Agree	Agree
Agree	Agree	Agree	Agree
Agree	Agree	Agree	Disagree
Strongly agree	Strongly agree	Strongly agree	Agree
Agree	Agree	Agree	Agree
Strongly agree	Strongly agree	Agree	Strongly agree
Agree	I do not know	Agree	Agree
Strongly agree	Strongly agree	Strongly agree	Agree
Agree	Agree	Agree	Agree
Strongly agree	Strongly agree	Strongly agree	Agree
Disagree	Disagree	I do not know	Disagree

I do not know	I do not know	Disagree	Disagree
Agree	I do not know	I do not know	Disagree
Agree	Strongly disagree	Strongly agree	I do not know
Agree	Strongly agree	Agree	Disagree
I do not know	I do not know	I do not know	I do not know
Strongly agree	Agree	Strongly agree	Strongly disagree
Agree	Agree	Agree	Agree
Disagree	Disagree	I do not know	Agree
Agree	Strongly agree	Agree	Agree
I do not know	I do not know	I do not know	Disagree
Agree	Agree	Agree	Agree
Agree	Agree	Agree	I do not know
Strongly agree	Agree	Agree	Strongly agree
Agree	I do not know	Agree	Strongly disagree
I do not know	I do not know	Agree	Agree
Agree	Agree	Agree	Disagree
Agree	I do not know	Strongly agree	Agree
I do not know	Agree	Agree	Agree
Agree	Agree	Agree	Agree
Agree	Agree	Agree	Agree
Agree	Agree	Agree	Agree
Agree	Agree	I do not know	I do not know
Strongly disagree	I do not know	Agree	Agree
Agree	Agree	Agree	Agree
Strongly agree	Strongly agree	Strongly agree	Disagree
Strongly agree	Agree	Strongly agree	Agree
Strongly agree	I do not know	Agree	Agree
I do not know	Strongly agree	I do not know	Agree
Agree	Agree	Agree	Agree
Agree	Disagree	Agree	Agree
Disagree	I do not know	I do not know	Disagree
Strongly agree	Strongly agree	Strongly agree	Strongly agree
Agree	Disagree	Agree	Agree
Strongly disagree	Strongly disagree	Strongly disagree	Agree
Agree	Strongly disagree	Agree	Disagree
Agree	I do not know	Strongly agree	Agree
Agree	I do not know	I do not know	I do not know
Agree	I do not know	I do not know	I do not know
Agree	Disagree	Agree	Agree
Disagree	Agree	Disagree	Disagree
Strongly agree	Disagree	Agree	Agree
Agree	Agree	Agree	Disagree
Agree	I do not know	Agree	Strongly agree
Agree	Agree	Agree	Agree
Disagree	Agree	Agree	Agree
Disagree	Agree	Agree	Agree
Agree	Strongly agree	Agree	Agree
Strongly disagree	Disagree	Disagree	Agree
Agree	Agree	Agree	I do not know
Agree	Agree	Disagree	Agree
Disagree	Disagree	Disagree	Agree
Agree	Disagree	Disagree	I do not know
Agree	Agree	Agree	Agree
Disagree	Disagree	Disagree	Disagree
Agree	Agree	Agree	Disagree

Strongly agree	Agree	Agree	Agree
Agree	I do not know	I do not know	I do not know
Agree	Strongly agree	Agree	Strongly agree
Strongly agree	Strongly agree	Agree	Agree
Strongly agree	Agree	Disagree	Strongly agree
Agree	I do not know	I do not know	Strongly agree
Strongly disagree	Agree	Strongly agree	Strongly agree
Strongly agree	Strongly agree	I do not know	Agree
I do not know	Strongly agree	I do not know	Agree
Strongly agree	I do not know	Agree	Disagree
Agree	Agree	Agree	Agree
I do not know	Agree	Agree	Agree
Agree	Agree	Agree	Strongly agree
Strongly agree	Agree	Strongly agree	Strongly agree
Agree	Agree	Agree	I do not know
Agree	I do not know	I do not know	Strongly disagree
Strongly agree	Strongly agree	Strongly agree	Strongly agree
Disagree	Strongly agree	Agree	Agree
Agree	Agree	I do not know	Disagree
Agree	Agree	Agree	Agree
I do not know	Agree	Agree	Agree
Strongly agree	Strongly agree	Strongly agree	Strongly agree
Strongly agree	Strongly agree	Agree	Agree
I do not know	Agree	Agree	Agree
Disagree	Disagree	Disagree	Agree
I do not know	I do not know	I do not know	Agree
Agree	I do not know	Disagree	Strongly disagree
Strongly disagree	Strongly disagree	Strongly disagree	Strongly disagree
Agree	Strongly agree	Agree	I do not know
Strongly disagree	Agree	Agree	Strongly disagree
Strongly agree	Strongly agree	Agree	Agree
Strongly agree	Agree	I do not know	Agree
Agree	Disagree	Agree	Disagree
Agree	Agree	Agree	Disagree
Agree	Agree	Agree	Agree
Agree	Agree	I do not know	Agree
Agree	Agree	Disagree	Strongly agree
I do not know	I do not know	I do not know	Disagree
Strongly agree	Strongly agree	Strongly agree	Strongly agree
Agree	Agree	Agree	Agree
I do not know	Agree	Agree	Agree
Agree	Disagree	Disagree	Disagree
Agree	Agree	Agree	Agree
Agree	Agree	Agree	Agree
I do not know	Disagree	I do not know	Disagree
Disagree	Disagree	Disagree	Agree
Agree	Agree	Agree	Agree
Disagree	Agree	Agree	Agree
Agree	Agree	Agree	Strongly agree
I do not know	Agree	I do not know	Strongly disagree
Agree	Agree	Agree	Agree
Strongly agree	Agree	Agree	Agree
Strongly disagree	Strongly disagree	Strongly disagree	Strongly disagree
Agree	Agree	Agree	Agree
Agree	Agree	Agree	Agree

Agree	Agree	Agree	Agree
Strongly agree	Strongly agree	Strongly agree	Strongly agree
Agree	Disagree	I do not know	Agree
Agree	Agree	Agree	Agree
Strongly agree	Strongly agree	I do not know	Agree
I do not know	I do not know	I do not know	Disagree
Agree	Strongly agree	Agree	I do not know
Strongly agree	Agree	Strongly agree	Strongly agree
I do not know	Agree	I do not know	I do not know
Agree	Strongly disagree	Disagree	Disagree
Agree	Strongly agree	Agree	I do not know
Agree	Agree	Agree	Disagree
Agree	Agree	Agree	Strongly agree
Agree	Disagree	Disagree	Disagree
Agree	Agree	Agree	Disagree
Agree	Agree	Agree	Disagree
Agree	Disagree	Disagree	Strongly disagree
Agree	Agree	Agree	Agree
Agree	Agree	Agree	Agree
Agree	Agree	Agree	Agree
Agree	Agree	Agree	Agree
Agree	Agree	Agree	Agree
Agree	Agree	Strongly agree	Strongly agree
Strongly agree	Strongly agree	Agree	Agree
Strongly agree	Strongly agree	Disagree	Strongly disagree
Disagree	Disagree	Disagree	Disagree
Agree	Agree	Agree	Agree
Strongly agree	Strongly agree	Strongly agree	Strongly agree
Agree	Agree	Strongly agree	Strongly agree
Agree	Strongly agree	Agree	Agree
Agree	Agree	Agree	Disagree
Agree	Agree	Agree	Agree
Agree	Agree	Agree	Strongly agree
I do not know	Agree	Disagree	Strongly agree
Agree	Agree	Agree	Agree
Strongly disagree	Agree	Strongly disagree	Agree
I do not know	Strongly agree	Agree	Strongly disagree
Agree	Agree	Agree	Strongly agree
Agree	Agree	Agree	Agree
Agree	Agree	I do not know	Agree
Agree	Agree	Agree	Agree
Strongly agree	Agree	Agree	Agree
Agree	Agree	Agree	Agree
Agree	Agree	Disagree	Disagree
Strongly disagree	Strongly disagree	Disagree	Strongly disagree
Disagree	Agree	Disagree	Agree
Agree	I do not know	I do not know	I do not know
Strongly agree	Strongly agree	Agree	Strongly agree
Strongly agree	Disagree	Disagree	Agree
I do not know	Agree	Agree	Disagree
Disagree	Agree	Agree	Agree
Agree	I do not know	Agree	Agree
I do not know	I do not know	Strongly agree	Strongly agree
Strongly agree	Disagree	Strongly disagree	Strongly disagree
Disagree	Disagree	Disagree	Disagree

Strongly agree	Strongly agree	Agree	Agree
Agree	Disagree	Disagree	Agree
I do not know	I do not know	I do not know	I do not know
Disagree	Agree	Strongly agree	Disagree
Agree	Strongly disagree	I do not know	Disagree
Agree	Agree	Agree	Agree
Disagree	Disagree	Agree	Disagree
Agree	Agree	Agree	Agree
Agree	Agree	Agree	Strongly agree
Agree	Agree	Agree	Agree
Disagree	Disagree	Agree	Disagree
Agree	Agree	Agree	Strongly agree
Strongly agree	Agree	Agree	Agree
Agree	Strongly agree	Agree	Strongly agree
Agree	Agree	Agree	Disagree
Strongly agree	Strongly agree	Strongly agree	Strongly agree
Strongly agree	Agree	Strongly agree	Agree
Agree	I do not know	Strongly disagree	Agree
Agree	Agree	Agree	Disagree
Agree	Agree	Disagree	Disagree
Strongly disagree	Agree	Strongly disagree	Agree
Strongly agree	Strongly agree	Strongly agree	Strongly agree
Disagree	I do not know	I do not know	Strongly disagree
Strongly disagree	I do not know	Strongly disagree	Strongly disagree
I do not know	Agree	I do not know	I do not know
Agree	Agree	Strongly agree	Strongly agree
Strongly agree	Strongly agree	Agree	Agree
Agree	Disagree	Agree	Agree
Agree	Agree	Strongly disagree	Strongly disagree
Strongly agree	Strongly agree	Agree	Strongly agree
Agree	Agree	Agree	Strongly agree
Agree	Agree	Agree	Agree
Agree	I do not know	I do not know	Agree
Agree	Agree	Agree	Agree
I do not know	Agree	Agree	Agree
Agree	Disagree	Agree	Agree
Agree	Agree	I do not know	Agree
Agree	Agree	Agree	I do not know
Agree	Disagree	Disagree	Agree
Agree	I do not know	Agree	Strongly agree
Disagree	Disagree	Agree	Disagree
Agree	Agree	Agree	Agree
Agree	Strongly agree	I do not know	Agree
Agree	I do not know	Agree	Agree
Agree	Agree	Agree	Agree
Strongly agree	Agree	Agree	Agree
Strongly agree	Strongly agree	Strongly agree	Strongly agree
Agree	Agree	Agree	Agree
Agree	Agree	I do not know	Disagree
Agree	Agree	Agree	I do not know
Agree	Disagree	Agree	Disagree
Disagree	Agree	Agree	Disagree
Strongly disagree	Strongly agree	I do not know	Strongly agree
Agree	Disagree	Agree	Strongly agree
Strongly agree	Agree	Disagree	Disagree

Strongly agree	Strongly agree	I do not know	Strongly agree
Strongly agree	Strongly agree	Agree	I do not know
Agree	Agree	Agree	Agree
Agree	Agree	Strongly agree	Strongly agree
Agree	Agree	Strongly agree	Strongly agree
I do not know	Strongly agree	Strongly agree	Agree
Disagree	Disagree	I do not know	I do not know
Disagree	Strongly agree	Strongly agree	Strongly agree
Agree	I do not know	Agree	Agree
Strongly agree	Strongly agree	Agree	Strongly agree
Agree	Agree	Agree	Agree
Agree	Agree	Agree	Agree
Strongly disagree	Strongly disagree	Strongly disagree	Agree
I do not know	I do not know	Agree	Agree
I do not know	Agree	I do not know	Agree
Agree	Strongly agree	Agree	Disagree
Disagree	Disagree	Agree	Agree
Agree	Strongly agree	Agree	Agree
Disagree	I do not know	I do not know	Disagree
Agree	Strongly agree	Disagree	Disagree
I do not know	Agree	Agree	Strongly agree
Agree	I do not know	I do not know	Agree
Agree	Agree	Strongly agree	Disagree
Disagree	Agree	Disagree	Strongly agree
Agree	Agree	I do not know	Agree
Agree	Strongly agree	Strongly agree	Agree
Agree	Agree	Agree	Strongly agree
Disagree	Disagree	Disagree	Disagree
Agree	Strongly agree	Strongly agree	Agree
Strongly agree	Strongly agree	Agree	Strongly agree
I do not know	Strongly agree	Strongly agree	Agree
Agree	Agree	Agree	Agree
I do not know	Strongly agree	Agree	Agree
Agree	I do not know	I do not know	Agree
Strongly agree	Strongly agree	Agree	Strongly agree
Agree	Agree	Agree	Strongly agree
Agree	Agree	Agree	Strongly agree
Disagree	Agree	Disagree	Strongly disagree
I do not know	Agree	I do not know	Strongly agree
Strongly agree	I do not know	Strongly agree	Strongly agree
Strongly agree	Strongly agree	Strongly agree	Strongly agree
Agree	Strongly disagree	Strongly agree	Disagree
Disagree	Disagree	I do not know	I do not know
I do not know	I do not know	I do not know	Disagree
Agree	Agree	Agree	Agree
Agree	I do not know	Disagree	Disagree
Agree	Strongly agree	Agree	Agree
Strongly agree	Strongly agree	Strongly agree	Strongly agree
Strongly agree	Agree	I do not know	Strongly agree
Strongly agree	Strongly agree	Agree	Strongly agree
I do not know	Agree	Agree	Agree
Strongly agree	I do not know	Agree	Disagree
Agree	Agree	Strongly agree	Strongly agree
Agree	Agree	Agree	Agree
Agree	Agree	Agree	Agree

Agree	Agree	I do not know	Strongly disagree
Agree	Disagree	Disagree	Disagree
I do not know	I do not know	I do not know	Disagree
Agree	Agree	Agree	Agree
Agree	Disagree	Agree	Disagree
Agree	I do not know	Agree	I do not know
Agree	Agree	Agree	Agree
Agree	Strongly agree	Agree	Strongly agree
I do not know	Agree	Agree	I do not know
I do not know	I do not know	Agree	Agree
Agree	Agree	Agree	Agree
Strongly agree	Strongly agree	Agree	Agree
Strongly agree	Agree	Agree	Agree
Agree	I do not know	Disagree	Agree
Agree	Strongly agree	Strongly agree	Strongly disagree
Agree	Agree	Disagree	Agree
Agree	Agree	Agree	Agree
Disagree	Agree	I do not know	Disagree
Disagree	Disagree	Disagree	Disagree
Agree	Agree	I do not know	I do not know
Strongly agree	Strongly agree	Agree	Strongly agree
Agree	Strongly agree	Agree	Agree
Agree	Strongly agree	Strongly agree	Disagree
Agree	Agree	Agree	Agree
Agree	Agree	Agree	Disagree
Strongly disagree	Strongly disagree	Agree	Disagree
Agree	Agree	Agree	Agree
Agree	Agree	I do not know	Agree
Agree	Agree	Agree	Agree
Disagree	I do not know	Disagree	Agree
Agree	Disagree	Agree	Disagree
Agree	Agree	Agree	I do not know
Strongly agree	Strongly agree	Strongly agree	Strongly agree
Agree	Agree	Agree	I do not know
Agree	Agree	Agree	Agree
I do not know	I do not know	I do not know	Disagree
Agree	Agree	Agree	Agree
Strongly agree	Strongly agree	Strongly agree	Strongly agree
Disagree	Agree	Agree	Agree
Disagree	Agree	Disagree	Disagree
I do not know	Agree	Agree	I do not know
Strongly disagree	I do not know	I do not know	I do not know
Agree	Agree	Agree	Agree
Agree	Agree	Agree	Agree
Agree	Agree	Agree	Agree
Agree	Agree	Agree	Agree
Strongly agree	Agree	Agree	I do not know
Agree	Agree	Agree	Agree
Agree	Strongly agree	Agree	Strongly agree
Agree	Agree	I do not know	I do not know
I do not know	Disagree	Disagree	Disagree
Agree	Strongly agree	Strongly agree	Strongly agree
Agree	Agree	Agree	Agree
Strongly agree	Agree	Agree	Agree
I do not know	Strongly disagree	Disagree	I do not know

Agree	Agree	Agree	Disagree
Strongly agree	Agree	Agree	Strongly agree
Agree	Agree	Agree	Agree
Disagree	Agree	Disagree	Strongly disagree
Disagree	Disagree	Disagree	Disagree
Agree	Disagree	Disagree	Agree
I do not know	Disagree	I do not know	Disagree
Agree	Agree	Strongly agree	Disagree
Strongly agree	Disagree	Agree	Disagree
Strongly disagree	Agree	Strongly disagree	Strongly disagree
Agree	Strongly agree	Disagree	Disagree
Agree	Disagree	Disagree	I do not know
I do not know	Agree	Disagree	Disagree
Strongly agree	Strongly agree	I do not know	Strongly disagree
Agree	Agree	I do not know	Agree
Strongly disagree	Agree	I do not know	I do not know
Agree	Agree	Agree	Agree
Agree	Agree	Agree	Agree
I do not know	Agree	Agree	Strongly agree
Agree	Agree	Agree	Agree
Strongly agree	Strongly agree	Strongly agree	Disagree
Disagree	Strongly disagree	I do not know	Strongly disagree
Agree	Agree	Agree	Strongly agree
Agree	Agree	Strongly agree	Disagree
Agree	Strongly agree	Agree	Agree
Disagree	Disagree	Agree	Agree
Agree	Agree	Agree	Agree
Strongly agree	Agree	Agree	Agree
Strongly agree	Agree	Agree	Disagree
Strongly disagree	Strongly agree	Strongly agree	I do not know
Agree	Agree	Agree	Strongly agree
Agree	Agree	Agree	Agree
Disagree	Agree	Disagree	Disagree
Agree	Agree	Disagree	Agree
Agree	Agree	Agree	Agree
Disagree	Disagree	Agree	Disagree
Agree	Agree	Agree	Agree
Disagree	Agree	Agree	Agree
Agree	Agree	Agree	Agree
Agree	Agree	Agree	Agree
Disagree	Disagree	Strongly disagree	Strongly disagree
Agree	Agree	Agree	Agree
I do not know	Agree	Agree	Agree
Strongly agree	Strongly agree	Strongly agree	Strongly agree
Agree	Agree	Strongly agree	Agree
Agree	Agree	Agree	Agree
I do not know	I do not know	Agree	Agree
Disagree	Disagree	Agree	Disagree
Agree	Agree	Agree	Strongly agree
Agree	Disagree	Agree	Strongly agree
I do not know	I do not know	Agree	Agree
Agree	Disagree	I do not know	I do not know
Agree	Agree	Agree	Agree
Strongly agree	Strongly agree	Strongly agree	Strongly agree
Agree	Disagree	Disagree	Agree

Agree	Agree	Agree	Agree
Disagree	Disagree	Disagree	Disagree
I do not know	Disagree	Agree	Agree
Strongly agree	Disagree	Strongly agree	Strongly disagree
Disagree	Agree	I do not know	Disagree
Agree	Agree	Strongly agree	Agree
Disagree	Disagree	Disagree	Disagree
Agree	Agree	Agree	I do not know
Agree	Agree	Agree	Agree
Strongly agree	Strongly agree	Strongly agree	Strongly agree
Agree	Disagree	Agree	Strongly disagree
Agree	Agree	Agree	I do not know
I do not know	Agree	I do not know	Agree
Strongly agree	Agree	Agree	Strongly agree
Agree	Agree	I do not know	Disagree
Agree	Disagree	I do not know	Disagree
Agree	Agree	I do not know	Agree

One of the reasons for viol	One of the reasons for viol	One of the reasons for viol	One of the reasons for viol
Agree	Agree	Agree	I do not know
Disagree	Strongly disagree	Disagree	Strongly disagree
Agree	Agree	Agree	Agree
Agree	Disagree	Agree	Disagree
Disagree	Disagree	Disagree	Disagree
Strongly agree	Agree	I do not know	Strongly agree
Disagree	Disagree	Agree	Disagree
Disagree	Disagree	Disagree	Disagree
Agree	Agree	Agree	Agree
Agree	I do not know	Agree	Agree
Disagree	Disagree	Agree	Disagree
Disagree	Strongly disagree	Disagree	Disagree
Agree	Disagree	Agree	Disagree
Strongly agree	Strongly agree	Agree	Agree
Strongly agree	Strongly agree	Strongly agree	Strongly agree
Agree	Agree	Agree	Agree
I do not know	I do not know	Agree	I do not know
Disagree	Disagree	Disagree	Agree
Strongly agree	Strongly agree	Strongly agree	Strongly agree
Disagree	I do not know	I do not know	I do not know
Agree	Strongly agree	Agree	Agree
Agree	I do not know	Agree	Agree
Strongly agree	Agree	Strongly agree	Strongly agree
Disagree	Disagree	Agree	Disagree
Disagree	Disagree	Agree	Disagree
Agree	Disagree	Agree	Agree
Agree	Agree	Disagree	I do not know
Disagree	Disagree	Disagree	Disagree
I do not know	Agree	Disagree	I do not know
Strongly agree	Agree	Agree	Strongly disagree
Disagree	Disagree	Agree	Disagree
Agree	I do not know	Disagree	I do not know
I do not know	I do not know	Agree	Disagree
Strongly agree	Agree	Strongly agree	Strongly agree
Strongly agree	Disagree	Agree	Strongly disagree
Strongly disagree	Strongly disagree	Strongly disagree	Strongly disagree
Strongly agree	Agree	Agree	Agree
Disagree	Disagree	Disagree	Disagree
Agree	Agree	Strongly agree	Agree
Strongly agree	Disagree	Agree	Disagree
Strongly agree	I do not know	Strongly agree	Strongly agree
Strongly agree	Strongly agree	Strongly agree	Strongly agree
Strongly agree	Strongly agree	Strongly agree	Strongly agree
I do not know	I do not know	Agree	I do not know
Agree	Disagree	Strongly agree	Agree
Strongly agree	I do not know	Strongly agree	Agree
Agree	Agree	Agree	Strongly agree
Agree	I do not know	Agree	Disagree

Agree	Agree	Strongly agree	Disagree
Disagree	Disagree	Disagree	Disagree
I do not know	I do not know	I do not know	I do not know
Agree	Disagree	Disagree	Disagree
Agree	Disagree	Disagree	Disagree
Agree	Disagree	Agree	Strongly agree
Agree	Disagree	Agree	Disagree
Agree	I do not know	Agree	Disagree
Agree	Disagree	Agree	I do not know
I do not know	Disagree	Agree	Agree
Agree	Disagree	Agree	Disagree
Strongly agree	Strongly agree	Strongly agree	Disagree
Agree	Disagree	Agree	Strongly disagree
I do not know	I do not know	Strongly disagree	Strongly disagree
Agree	Disagree	Agree	Disagree
Agree	Agree	Agree	Agree
Agree	Agree	Agree	Agree
Agree	Agree	Strongly agree	Strongly disagree
Strongly agree	Agree	Agree	Agree
Agree	Agree	Agree	Agree
Agree	Agree	I do not know	I do not know
Agree	I do not know	Agree	Disagree
Agree	Disagree	I do not know	Disagree
Agree	Agree	Agree	Agree
Agree	I do not know	Agree	Agree
Strongly agree	I do not know	Strongly agree	Disagree
I do not know	Strongly disagree	Strongly agree	Disagree
Strongly disagree	Agree	Agree	I do not know
Agree	Agree	Agree	Agree
I do not know	Disagree	I do not know	Disagree
Strongly agree	Agree	Agree	Agree
Disagree	Disagree	Disagree	Disagree
Agree	Agree	Agree	Agree
Disagree	Disagree	Agree	Disagree
Agree	Strongly agree	Agree	Agree
Agree	Strongly agree	Strongly agree	Agree
Agree	Agree	Agree	Agree
Disagree	I do not know	Agree	Agree
Agree	Agree	Agree	Agree
Agree	Disagree	Agree	Disagree
Strongly agree	Strongly agree	Strongly agree	Agree
I do not know	Agree	Strongly disagree	Strongly disagree
Agree	I do not know	I do not know	I do not know
Agree	I do not know	Agree	Agree
I do not know	Agree	Agree	I do not know
Agree	Agree	Agree	Agree
Disagree	Disagree	Agree	Agree
Strongly agree	Strongly agree	Agree	Strongly agree
I do not know	I do not know	Agree	I do not know
Strongly agree	Agree	Strongly agree	Strongly agree
I do not know	Strongly agree	Agree	Strongly agree
Agree	Strongly agree	Strongly agree	Strongly agree
I do not know	Agree	Agree	I do not know
Strongly agree	Agree	Strongly agree	Strongly agree
Disagree	Disagree	I do not know	Disagree

Disagree	Strongly disagree	Strongly disagree	Strongly disagree
Disagree	Disagree	Disagree	Disagree
Strongly disagree	Strongly disagree	I do not know	Strongly disagree
Agree	Agree	Agree	Strongly agree
I do not know	Agree	I do not know	I do not know
Strongly agree	Disagree	Agree	Strongly agree
Agree	Agree	Agree	Agree
Strongly disagree	Disagree	Strongly disagree	Strongly agree
Agree	Agree	Agree	I do not know
Agree	I do not know	Strongly agree	I do not know
Agree	Disagree	I do not know	I do not know
I do not know	Agree	Agree	Disagree
Agree	Agree	Agree	Agree
Disagree	Disagree	Disagree	Disagree
Agree	Agree	Agree	I do not know
Agree	Disagree	Disagree	Disagree
Agree	Disagree	I do not know	Agree
Agree	I do not know	Agree	I do not know
Agree	Agree	Agree	Agree
Agree	I do not know	Agree	Agree
Agree	Agree	Agree	Disagree
Agree	Agree	Strongly agree	I do not know
Agree	I do not know	Agree	Agree
Agree	I do not know	Agree	Agree
Strongly agree	Disagree	Strongly agree	Disagree
Agree	I do not know	Agree	Agree
Agree	Agree	Strongly agree	Strongly disagree
Strongly agree	Agree	Agree	Agree
I do not know	I do not know	Agree	I do not know
Agree	I do not know	Agree	I do not know
Disagree	I do not know	Disagree	Disagree
Strongly agree	Strongly agree	Strongly agree	Strongly agree
Agree	Disagree	Agree	Disagree
Agree	I do not know	Strongly disagree	Strongly disagree
Disagree	Disagree	Disagree	Strongly agree
Strongly agree	I do not know	Agree	Disagree
Strongly agree	Strongly disagree	Disagree	Disagree
Strongly agree	Strongly disagree	Disagree	Disagree
Disagree	Disagree	Agree	I do not know
Disagree	Disagree	Agree	Agree
Disagree	Disagree	Agree	Strongly disagree
Disagree	Disagree	Disagree	Disagree
Agree	Agree	Strongly agree	Agree
Agree	Agree	Agree	Agree
Agree	Disagree	Agree	Agree
Agree	Agree	Agree	Agree
Agree	Agree	Agree	Disagree
Agree	Disagree	Agree	Disagree
Agree	Agree	Strongly agree	Strongly agree
Agree	Agree	Agree	Agree
Disagree	Agree	Agree	Agree
Strongly agree	Disagree	Strongly agree	Strongly agree
Agree	Agree	Agree	Agree
Disagree	Disagree	Disagree	Disagree
Disagree	Disagree	Agree	Disagree

Disagree	Disagree	Disagree	Strongly disagree
I do not know	Agree	Agree	I do not know
Agree	I do not know	Agree	Agree
Agree	Strongly agree	Strongly agree	Strongly agree
Agree	Strongly disagree	Strongly agree	Agree
Agree	Agree	Strongly agree	Agree
Strongly agree	Agree	Strongly agree	Strongly agree
Strongly agree	Strongly agree	Strongly agree	Agree
Strongly disagree	Strongly agree	Agree	Strongly agree
Disagree	Disagree	Disagree	Disagree
Agree	Agree	Agree	Strongly disagree
I do not know	I do not know	Disagree	Disagree
Strongly agree	Disagree	Agree	Disagree
Strongly agree	Strongly agree	Strongly agree	Agree
Agree	I do not know	Agree	I do not know
Strongly disagree	Disagree	Disagree	Disagree
Strongly agree	Strongly agree	Strongly agree	Strongly agree
Agree	Agree	Agree	Agree
Disagree	Disagree	Agree	Disagree
Agree	Agree	I do not know	Agree
Agree	I do not know	I do not know	Agree
Strongly agree	Strongly agree	Strongly agree	Strongly agree
Agree	Agree	Agree	Disagree
Agree	Agree	Agree	I do not know
Agree	Disagree	I do not know	Disagree
I do not know	Disagree	Agree	Disagree
Agree	Disagree	Agree	Strongly disagree
Strongly disagree	Strongly disagree	Strongly disagree	Strongly disagree
Strongly agree	I do not know	Agree	Strongly agree
Strongly disagree	Strongly disagree	Strongly disagree	Agree
Agree	Strongly agree	Strongly agree	Strongly agree
Agree	Disagree	Agree	Disagree
Disagree	Agree	Agree	Disagree
Agree	Disagree	Disagree	Disagree
Agree	Agree	Strongly agree	Agree
Agree	Disagree	Disagree	Disagree
Strongly agree	Strongly agree	Strongly agree	Strongly disagree
Disagree	Disagree	Strongly disagree	I do not know
Strongly agree	Strongly agree	Strongly agree	Strongly agree
Agree	Disagree	Agree	Disagree
Agree	Agree	Agree	Disagree
Agree	Disagree	Disagree	Disagree
Agree	Agree	Agree	Disagree
Agree	I do not know	Agree	Agree
Disagree	Disagree	Disagree	Disagree
Agree	Disagree	Agree	Disagree
Agree	Agree	Agree	Agree
Agree	Disagree	Agree	Agree
Strongly agree	Agree	Strongly agree	Strongly agree
Strongly disagree	Strongly disagree	I do not know	I do not know
Agree	I do not know	Agree	Agree
Agree	Agree	Strongly agree	Strongly agree
Strongly disagree	Strongly disagree	Strongly disagree	I do not know
Agree	Disagree	Agree	Agree
Agree	Strongly disagree	Agree	Strongly disagree

Agree	Agree	Agree	Agree
Strongly agree	Strongly agree	Strongly agree	I do not know
I do not know	Agree	Agree	Disagree
Agree	Agree	Strongly agree	Strongly agree
Agree	I do not know	Strongly agree	I do not know
I do not know	I do not know	I do not know	I do not know
Agree	Disagree	I do not know	Disagree
Strongly agree	I do not know	Strongly agree	Strongly agree
Agree	Strongly agree	Agree	Strongly disagree
Agree	Disagree	Disagree	Disagree
I do not know	Disagree	Agree	Disagree
Disagree	Disagree	Disagree	Disagree
Strongly agree	Agree	Strongly agree	Strongly agree
Disagree	Disagree	Disagree	Disagree
Agree	Disagree	Agree	Disagree
Disagree	Disagree	Agree	Strongly agree
Disagree	Strongly disagree	Agree	Strongly disagree
Agree	Agree	Agree	Agree
Agree	I do not know	Agree	I do not know
I do not know	Disagree	Strongly agree	I do not know
Agree	I do not know	I do not know	I do not know
Agree	Agree	Agree	Agree
Strongly agree	Strongly agree	Strongly agree	Strongly agree
Agree	Agree	Agree	Agree
I do not know	Strongly disagree	Agree	Strongly disagree
Disagree	Disagree	Disagree	Disagree
Agree	Disagree	Disagree	Agree
Strongly agree	Strongly agree	Strongly agree	Strongly agree
Agree	I do not know	I do not know	Agree
Agree	I do not know	Disagree	Agree
I do not know	Disagree	Agree	Disagree
Agree	Agree	Agree	Agree
Agree	Disagree	I do not know	Strongly disagree
Strongly agree	Disagree	Strongly agree	Strongly agree
Agree	Agree	Agree	Agree
Agree	I do not know	Strongly disagree	Strongly disagree
Agree	I do not know	Agree	Disagree
Strongly agree	I do not know	Strongly agree	Strongly agree
Agree	Agree	Agree	Agree
Agree	Disagree	Agree	Agree
Agree	Agree	Agree	Agree
Strongly agree	Agree	Strongly agree	Agree
Agree	Agree	Agree	Agree
Strongly disagree	Strongly disagree	Disagree	Strongly disagree
Strongly disagree	Strongly disagree	Disagree	Strongly disagree
Strongly agree	Disagree	Strongly agree	Agree
I do not know	Disagree	Agree	I do not know
Strongly agree	Agree	Agree	Agree
Agree	I do not know	Agree	Agree
Agree	I do not know	Agree	Disagree
Agree	Disagree	Strongly disagree	Agree
Agree	Agree	Agree	Agree
Strongly agree	I do not know	I do not know	Strongly agree
I do not know	Disagree	Disagree	Disagree
Disagree	Disagree	Disagree	Disagree

Agree	Agree	Agree	Agree
Agree	Agree	Disagree	Agree
I do not know	I do not know	I do not know	I do not know
Agree	Agree	Agree	Agree
Agree	I do not know	I do not know	I do not know
Agree	Agree	Agree	Agree
Agree	Disagree	Disagree	Disagree
Agree	Strongly agree	Strongly agree	Strongly agree
Agree	Agree	Agree	Agree
Agree	Agree	Agree	Agree
Disagree	Strongly disagree	Disagree	Strongly agree
Strongly agree	Agree	Strongly agree	Agree
Agree	Agree	Agree	Disagree
Strongly agree	Strongly agree	Strongly agree	Strongly agree
Agree	Disagree	Strongly agree	Disagree
Strongly agree	Strongly agree	Strongly agree	Strongly agree
Strongly agree	Strongly agree	Agree	Agree
Agree	Agree	Strongly disagree	Agree
Agree	Agree	Agree	Agree
Disagree	Disagree	Disagree	Disagree
Strongly agree	Agree	Agree	Agree
Strongly agree	Strongly agree	Strongly agree	Strongly agree
I do not know	Strongly disagree	Strongly disagree	Strongly disagree
Disagree	Disagree	Disagree	Disagree
Agree	I do not know	I do not know	I do not know
Strongly agree	Strongly agree	Strongly agree	Strongly agree
Agree	Agree	Agree	Agree
Agree	Agree	Agree	Disagree
Agree	Agree	Agree	Disagree
Agree	Agree	Strongly agree	Agree
Agree	Disagree	Agree	Disagree
Agree	Disagree	Agree	Disagree
Agree	I do not know	Agree	I do not know
Agree	Agree	Agree	I do not know
I do not know	I do not know	I do not know	I do not know
Agree	Agree	Strongly agree	Disagree
Agree	Strongly disagree	Agree	I do not know
Agree	Agree	I do not know	Disagree
Agree	Agree	Agree	Disagree
Strongly agree	Strongly agree	Agree	Agree
Disagree	Disagree	Disagree	Disagree
Agree	Agree	Strongly agree	Disagree
Disagree	Disagree	Agree	Agree
Agree	Agree	Agree	Agree
Agree	Agree	Agree	Agree
Strongly agree	I do not know	Strongly agree	Strongly agree
Strongly agree	Strongly agree	Strongly agree	Strongly agree
Agree	I do not know	Agree	I do not know
Disagree	Agree	Strongly disagree	Agree
Disagree	Disagree	Agree	Disagree
Agree	Disagree	Disagree	Agree
Agree	Disagree	I do not know	Strongly agree
Strongly agree	Strongly agree	Agree	Agree
Strongly agree	Agree	Strongly agree	Disagree
Strongly disagree	Strongly disagree	Disagree	Disagree

Agree	I do not know	Agree	Agree
Strongly agree	Strongly agree	Strongly agree	Strongly agree
Agree	Agree	Agree	Agree
Strongly agree	Agree	Strongly agree	Disagree
Agree	I do not know	Agree	Disagree
Agree	Agree	Agree	Agree
Agree	Disagree	I do not know	Agree
Strongly agree	Strongly agree	Strongly agree	Strongly agree
Agree	I do not know	Agree	Agree
Agree	Strongly agree	Strongly agree	Strongly agree
Agree	Agree	Agree	I do not know
Agree	Agree	Agree	Agree
I do not know	Agree	Strongly disagree	I do not know
Agree	Strongly agree	Agree	Strongly agree
Disagree	Disagree	Agree	Agree
Agree	Disagree	Strongly agree	Agree
Agree	I do not know	Agree	Agree
Agree	Agree	Agree	Agree
I do not know	Strongly disagree	I do not know	Agree
Disagree	Disagree	Strongly agree	I do not know
Agree	Agree	Agree	Strongly disagree
Agree	I do not know	Disagree	I do not know
I do not know	Strongly agree	Agree	Agree
Disagree	Disagree	Agree	Agree
I do not know	Disagree	Disagree	Disagree
Agree	Agree	Agree	Agree
Strongly agree	Strongly agree	Agree	Agree
Agree	Agree	Agree	Disagree
Agree	Agree	Strongly agree	Disagree
Agree	Agree	Strongly agree	Strongly agree
Strongly agree	Strongly agree	Agree	Agree
Agree	Agree	Agree	Agree
Agree	I do not know	Strongly agree	Agree
Agree	I do not know	Agree	Agree
Strongly agree	I do not know	Strongly agree	Strongly agree
Strongly agree	Agree	Strongly agree	Strongly agree
Strongly agree	Agree	Strongly agree	Strongly agree
I do not know	Strongly disagree	Strongly disagree	Strongly disagree
Agree	I do not know	Agree	Agree
Strongly agree	Strongly agree	Strongly agree	Strongly agree
Agree	Strongly agree	I do not know	Strongly agree
Agree	Disagree	Agree	Agree
Disagree	Disagree	I do not know	I do not know
Agree	Agree	I do not know	Agree
Agree	Agree	Agree	Agree
Agree	Disagree	Agree	Disagree
I do not know	Disagree	Strongly agree	Disagree
Strongly agree	Strongly agree	Strongly agree	Strongly agree
Strongly agree	Agree	Strongly agree	Agree
Strongly agree	I do not know	Agree	Strongly agree
Agree	Agree	Agree	I do not know
Disagree	Agree	Agree	Disagree
Agree	Agree	Agree	Disagree
Agree	Agree	Agree	Agree
Agree	Agree	Agree	Disagree

Strongly disagree	Agree	Agree	Strongly agree
Disagree	Disagree	Agree	Disagree
I do not know	Disagree	I do not know	I do not know
Strongly agree	Agree	Agree	Strongly agree
Agree	Disagree	Agree	Disagree
Agree	I do not know	I do not know	Strongly disagree
Agree	Agree	Agree	Agree
Agree	Agree	Agree	Strongly agree
Agree	I do not know	Agree	I do not know
Agree	Agree	Agree	Strongly agree
Agree	I do not know	Strongly agree	Strongly agree
Agree	I do not know	Strongly agree	Strongly agree
Agree	Agree	Agree	Strongly disagree
Agree	Disagree	Agree	Strongly disagree
Strongly agree	Strongly agree	Strongly agree	Strongly agree
Agree	I do not know	Agree	Agree
Agree	Agree	Agree	Agree
Disagree	Disagree	I do not know	I do not know
I do not know	Disagree	Disagree	Disagree
I do not know	I do not know	I do not know	Agree
Strongly agree	Agree	Strongly agree	I do not know
Strongly agree	I do not know	Agree	Strongly disagree
Agree	Agree	Agree	Disagree
I do not know	I do not know	Agree	Disagree
Disagree	Disagree	I do not know	I do not know
Disagree	Disagree	Disagree	Disagree
Agree	Agree	Agree	Agree
Agree	Agree	Agree	Agree
Agree	Strongly agree	Strongly agree	Strongly agree
Agree	Agree	Disagree	Disagree
Agree	Disagree	Agree	Disagree
Agree	Agree	Agree	Agree
Strongly agree	Strongly agree	Strongly agree	Strongly agree
Agree	I do not know	I do not know	I do not know
Agree	Disagree	Agree	Disagree
Agree	I do not know	Agree	Agree
Agree	Agree	Agree	Agree
Strongly agree	Strongly agree	Strongly agree	Strongly agree
Agree	Agree	I do not know	Strongly disagree
Disagree	Disagree	Strongly disagree	Disagree
Agree	Agree	Agree	Agree
I do not know	I do not know	I do not know	I do not know
Agree	Agree	Agree	I do not know
I do not know	Disagree	I do not know	Agree
Agree	Agree	Strongly agree	Strongly agree
Agree	Agree	Agree	Agree
I do not know	Agree	Agree	Agree
Agree	Agree	I do not know	Agree
Agree	I do not know	Agree	Strongly agree
Agree	I do not know	Agree	Disagree
Disagree	Disagree	Disagree	Disagree
Strongly disagree	Strongly agree	I do not know	Strongly agree
Agree	I do not know	Strongly agree	I do not know
Strongly agree	Agree	Agree	Disagree
Strongly disagree	Strongly agree	Agree	I do not know

Strongly disagree	Disagree	Disagree	Agree
Strongly agree	Strongly agree	Strongly agree	Strongly agree
Agree	Agree	Agree	Agree
Disagree	I do not know	Disagree	Strongly disagree
Disagree	Disagree	Agree	Disagree
Agree	Agree	Agree	Agree
Disagree	Disagree	Disagree	Disagree
Agree	Disagree	Disagree	Agree
Agree	Disagree	Strongly agree	Disagree
I do not know	Strongly disagree	Agree	I do not know
Strongly disagree	Disagree	Disagree	Disagree
Agree	Disagree	I do not know	Disagree
Agree	Strongly agree	Agree	Strongly agree
Strongly disagree	Strongly disagree	Strongly disagree	Strongly disagree
Agree	I do not know	Agree	I do not know
Disagree	Disagree	Agree	I do not know
Agree	Agree	Agree	Agree
Agree	Agree	Disagree	Strongly disagree
Strongly agree	I do not know	Strongly agree	Agree
I do not know	Agree	Agree	Agree
Agree	Agree	Strongly agree	Strongly disagree
Strongly disagree	Strongly disagree	Strongly disagree	Disagree
Strongly agree	Strongly agree	Strongly agree	Agree
I do not know	Agree	Agree	Strongly agree
Agree	Agree	Agree	Agree
Disagree	Disagree	Disagree	Agree
Agree	Agree	Agree	Agree
Agree	Agree	Strongly agree	Agree
I do not know	Strongly agree	Strongly agree	Agree
Strongly agree	Strongly disagree	Agree	Agree
Strongly agree	Strongly disagree	Disagree	Disagree
Agree	Agree	Agree	Agree
Agree	Agree	Agree	Disagree
Agree	Disagree	Agree	I do not know
Agree	I do not know	Strongly agree	Agree
Disagree	Agree	Agree	I do not know
Agree	I do not know	I do not know	I do not know
Agree	Agree	Disagree	Agree
Agree	Agree	Agree	Agree
Agree	Agree	Agree	Disagree
Disagree	Strongly disagree	Disagree	I do not know
Strongly disagree	Agree	Agree	I do not know
Disagree	Agree	Agree	Agree
Strongly agree	Strongly agree	Strongly agree	Strongly agree
Agree	Strongly agree	Agree	Agree
Agree	Agree	Agree	Agree
Agree	I do not know	Agree	I do not know
Disagree	Disagree	Disagree	Disagree
Strongly agree	Agree	Strongly agree	Agree
Strongly agree	Disagree	Strongly agree	Strongly disagree
Agree	Agree	Agree	Agree
Disagree	Agree	Disagree	I do not know
Strongly agree	Agree	Agree	Strongly agree
Strongly agree	Strongly agree	Strongly agree	Strongly agree
Disagree	Disagree	Agree	Agree

I do not know	I do not know	Agree	Disagree
Disagree	Disagree	Disagree	Disagree
Agree	Agree	Agree	Strongly disagree
Strongly disagree	Strongly disagree	Strongly disagree	Strongly disagree
I do not know	Agree	Disagree	I do not know
Strongly agree	Agree	Agree	Disagree
Disagree	Disagree	I do not know	Disagree
Agree	I do not know	Agree	Agree
Agree	Agree	Agree	Agree
Strongly agree	Strongly agree	Strongly agree	Strongly agree
Agree	Agree	Disagree	I do not know
I do not know	I do not know	I do not know	I do not know
Agree	Disagree	Agree	Disagree
Strongly agree	Agree	Agree	Agree
Agree	Agree	Agree	Agree
I do not know	I do not know	Disagree	Disagree
Agree	I do not know	I do not know	I do not know

Have you ever been partici	Have you ever been participated in an act of verbal violence against healthcare w
No	No
No	No
No	No
No	No
No	No
No	No
No	No
	No
No	No
No	No
No	Yes
No	No
No	No
Yes	Yes
No	Yes
No	No
No	No
No	No
No	No
No	No
No	No
No	No
No	No
No	No
No	No
No	No
No	No
No	No
No	No
No	No
No	No
No	No
Yes	Yes
No	No
No	No
No	No
No	No
No	No
No	Yes
No	No
No	No
No	No
No	Yes
No	No
No	Yes
No	No
No	Yes
No	Yes

No	No
No	No
No	No
No	No
No	No
No	Yes
No	Yes
No	No
No	No
No	No
No	No
Yes	Yes
No	Yes
No	No
No	No
No	No
No	No
No	No
No	No
No	No
No	Yes
No	No
No	No
No	No
No	No
No	No
No	No
No	No
No	Yes
No	No
No	No
No	Yes
No	No
No	No
No	No
No	No
No	No
No	No
No	No
No	Yes
No	No
No	No
Yes	Yes
No	No
No	No
No	No
No	Yes
Yes	Yes
No	No
No	No
Yes	Yes
No	Yes
No	No
No	Yes
No	No

No	No
No	No
No	No
No	No
No	No
No	No
No	Yes
No	No
No	No
No	No
No	No
No	No
Yes	Yes
No	No
No	No
No	No
No	Yes
No	No
No	No
No	Yes
No	No
No	No
No	No
No	No
Yes	No
No	Yes
No	No
No	No
No	No
No	No
No	No
No	Yes
No	No
No	No
Yes	Yes
No	No
No	No
No	No
No	No
No	No
No	No
No	No
No	No
No	No
No	Yes
No	No
Yes	Yes
No	No
No	No
No	No
No	No
No	No
No	No
No	No
No	No

No	No
No	Yes
No	No
No	No
No	No
No	No
No	No
No	No
No	No
No	No
No	No
No	Yes
No	No
No	No
Yes	No
No	No
No	No
No	Yes
No	No
No	
No	No
No	No
No	No
No	No
No	No
No	No
No	No
No	No
No	No
No	No
No	Yes
No	No
No	No
No	Yes
No	Yes
Yes	Yes
No	No
Yes	Yes
No	No
No	No
No	Yes
No	No
No	No
No	No
No	No
No	No
No	No
No	Yes
No	Yes
No	No
No	Yes
No	No
No	No
No	No

No	No
No	
No	No
No	No
No	Yes
No	No
No	No
No	Yes
No	No
No	No
No	No
No	No
No	No
No	No
No	Yes
No	No
No	No
No	No
No	Yes
Yes	Yes
No	No
No	No
No	No
No	No
No	No
No	No
No	No
No	No
No	No
No	No
No	Yes
No	No
No	No
No	No
No	No
No	No
No	No
No	No
No	Yes
No	No
No	No
No	No
No	No
No	No
No	No
Yes	No
No	No
No	No
No	No
No	No
No	No
No	No
No	Yes
No	No
No	No

Yes	No
No	No
No	No
No	No
No	No
No	No
No	No
Yes	Yes
No	No
No	No
No	Yes
No	No
No	Yes
No	No
No	No
Yes	No
No	No
No	No
No	No
No	No
No	No
No	Yes
No	No
No	No
No	No
No	No
No	No
No	No
No	No
No	No
No	Yes
No	No
No	No
No	No
No	No
No	No
No	No
No	No
No	No
No	No
No	No
No	Yes
No	No
No	No
No	No
No	No
No	No
No	No
No	No
No	No
No	No
No	No
No	No
No	No
No	Yes

No	No
No	Yes
No	No
No	Yes
Yes	Yes
No	No
No	No
No	Yes
No	No
No	No
No	No
No	No
No	No
No	No
No	No
Yes	No
No	No
No	No
No	No
No	No
No	No
No	No
No	No
No	No
No	No
No	No
No	Yes
No	No
No	No
No	Yes
No	No
No	No
No	Yes
No	No
No	No
No	No
Yes	Yes
No	Yes
No	Yes
No	Yes
No	No
No	No
No	No
No	No
No	No
No	No
No	No
Yes	Yes
No	Yes
Yes	No
No	No
No	Yes
Yes	Yes
No	No
No	No

No	Yes
No	No
No	No
No	No
No	No
No	Yes
No	Yes
Yes	Yes
No	No
No	No
No	No
No	No
No	No
Yes	Yes
Yes	Yes
No	Yes
No	No
No	No
No	No
No	No
No	No
No	No
No	No
No	No
No	No
No	No
No	No
No	No
No	No
No	No
No	Yes
No	No
Yes	Yes
No	No
No	Yes
No	No
No	No
Yes	Yes
No	No
No	No
No	No
No	No
No	No
No	No
No	No
No	Yes
No	No
No	No
No	No
No	No
No	No
Yes	No
No	No
No	No
No	No

No	No
No	No
No	No
No	No
No	No
No	No
No	No
No	No
No	Yes
No	
No	No
No	Yes
No	No
Yes	Yes
No	No
No	No
No	No
No	No
No	No
No	No
No	No
No	No
No	No
No	No
No	Yes
No	No
Yes	Yes
Yes	Yes
No	No
Yes	No
No	No
No	No
No	No
No	No
No	Yes
No	No
No	No
No	No
No	No
No	No
No	No
No	No
No	Yes
Yes	No
No	No
No	No
No	No
No	Yes
No	No
No	No
No	No
Yes	No
No	Yes
No	Yes
No	No

No	No
No	Yes
No	No
No	No
No	No
No	No
No	No
No	No
No	No
No	No
No	No
No	No
No	No
No	No
No	No
No	No
No	No

workers?
