# Supplementary material for: Breaching the Bridge: An Investigation into Doctor-Patient Miscommunication as a Significant Factor in the Violence against Healthcare Workers in Palestine
Source: Biomed Res Int. 2021 Jul 23;2021:9994872. doi: 10.1155/2021/9994872 (PMC8324345; doi:10.1155/2021/9994872)
Supplement: Supplementary Materials — All the utilized data to support the findings of the current study are included in the supplementary material. [file 9994872.f1.zip › TOOL.docx]

**Questionnaire**

**“Breaching the Bridge: An investigation into doctor-patient miscommunication as a significant factor in the violence against**

**medical practitioners in Palestine”.**

Dear Prospective Participant,

The researchers, Munther Saeedi, Dr. Nihad Al-Othman and ________ , from An-Najah University are conducting a research entitled “Breaching the Bridge: An investigation into doctor-patient miscommunication as a significant factor in the violence against medical practitioners in Palestine”. The purpose of this research is to find out to what extent poor communication on the part of medical practitioners can be a real cause for attacks on them. It also seeks to come up with some possible solutions that may help boost medical practitioners’ communication skills to avoid such problems. We assure you, dear respondent, that your responses to the items in this questionnaire are to be used for scientific purposes only and they are to be kept secret.

Demographic Data:

| Name: | Gender: Male/ Female |
| --- | --- |
| Age: | Place of residence |
| 30 or less | City: |
| 40 or less | Village: |
| 50 or less | Refugee camp |
| 60 or less |  |
| Education: |  |
| Diploma or less |  |
| BA |  |
| High Studies |  |

| ***Verbal communication:*** | Strongly agree | Agree | I don't  Know | Disagree | Strongly  disagree |
| --- | --- | --- | --- | --- | --- |
| 1 . One of the reasons for violence, physical or verbal, against medical practitioners is because they don't use simplified, clear language. |  |  |  |  |  |
| 2. One of the reasons for violence, physical or verbal, against medical practitioners is because they don't consider patients and their relatives' educational level. |  |  |  |  |  |
| 3. One of the reasons for violence, physical or verbal, against medical practitioners is because they don't speak clearly when they communicate with patients and their relatives. |  |  |  |  |  |
| 4. One of the reasons for violence, physical or verbal, against medical practitioners is because they don’t take into consideration the psychological status of patients and their relatives. |  |  |  |  |  |
| 5. One of the reasons for violence, physical or verbal, against medical practitioners is because they don’t pick the right time to break bad news. |  |  |  |  |  |
| 6. One of the reasons for violence, physical or verbal, against medical practitioners is because they don’t answer patients and relatives' questions well. |  |  |  |  |  |
| 7. One of the reasons for violence, physical or verbal, against medical practitioners is because they show some superiority when communicating with patients and relatives. |  |  |  |  |  |
| 8. One of the reasons for violence, physical or verbal, against medical practitioners is because they don’t show sympathy and empathy when communicating with patients and relatives. |  |  |  |  |  |
| 9. One of the reasons for violence, physical or verbal, against medical practitioners is because they don’t show much concentration when communicating with patients and relatives. |  |  |  |  |  |
| 10. One of the reasons for violence, physical or verbal, against medical practitioners is because they don't use courteous language when communicating with patients and relatives. |  |  |  |  |  |
| 11. One of the reasons for violence, physical or verbal, against medical practitioners is because they aren't competent enough to ask the right questions when communicating with patients and relatives. |  |  |  |  |  |
| 12. One of the reasons for violence, physical or verbal, against medical practitioners is because they don’t listen attentively when communicating with patients and relatives. |  |  |  |  |  |
| 13. One of the reasons for violence, physical or verbal, against medical practitioners is because they can't handle patients and relatives' complaints appropriately. |  |  |  |  |  |
| 14. One of the reasons for violence, physical or verbal, against medical practitioners is because they aren't competent enough to ask open-ended questions to enable patients and their relatives to speak freely.  ***Non-verbal communication:*** |  |  |  |  |  |
|  | Strongly agree | Agree | I don't  Know | Disagree | Strongly  disagree |
| 1 . One of the reasons for violence, physical or verbal, against medical practitioners is because they don’t maintain good eye contact. |  |  |  |  |  |
| 2. One of the reasons for violence, physical or verbal, against medical practitioners is because they don’t smile frequently. |  |  |  |  |  |
| 3. One of the reasons for violence, physical or verbal, against medical practitioners is because they don’t a comfortable voice tone. |  |  |  |  |  |
| 4. One of the reasons for violence, physical or verbal, against medical practitioners is because they often have a frown on their faces. |  |  |  |  |  |
| 5. One of the reasons for violence, physical or verbal, against medical practitioners is because they are often seated in a provocative manner. |  |  |  |  |  |
| 6. One of the reasons for violence, physical or verbal, against medical practitioners is because they don’t employ handshake properly. |  |  |  |  |  |
| ***Part Four:*** |  |  |  |  |  |
|  | Strongly agree | Agree | I don't  Know | Disagree | Strongly  disagree |
| 1 . Have you ever been involved in an act of physical violence against medical practitioners? |  |  |  |  |  |
| 2. Have you ever been involved in an act of verbal violence against medical practitioners? |  |  |  |  |  |
